# Applying contrastive pre-training for depression and anxiety risk prediction in type 2 diabetes patients based on heterogeneous electronic health records: a primary healthcare case study

**DOI:** 10.1093/jamia/ocad228

**Published:** 2023-12-07

**Authors:** Wei Feng, Honghan Wu, Hui Ma, Zhenhuan Tao, Mengdie Xu, Xin Zhang, Shan Lu, Cheng Wan, Yun Liu

**Affiliations:** Department of Medical Informatics, School of Biomedical Engineering and Informatics, Nanjing Medical University, Nanjing, Jiangsu, 210009, China; Institute of Health Informatics, University College London, London, WC1E 6BT, United Kingdom; The Alan Turing Institute, London, NW1 2DB, United Kingdom; Department of Medical Psychology, Nanjing Brain Hospital affiliated with Nanjing Medical University, Nanjing, Jiangsu, 210024, China; Department of Planning, Nanjing Health Information Center, Nanjing, Jiangsu, 210003, China; Department of Medical Informatics, School of Biomedical Engineering and Informatics, Nanjing Medical University, Nanjing, Jiangsu, 210009, China; Department of Medical Informatics, School of Biomedical Engineering and Informatics, Nanjing Medical University, Nanjing, Jiangsu, 210009, China; Department of Information, The First Affiliated Hospital, Nanjing Medical University, Nanjing, Jiangsu, 210029, China; Department of Medical Informatics, School of Biomedical Engineering and Informatics, Nanjing Medical University, Nanjing, Jiangsu, 210009, China; Department of Information, The First Affiliated Hospital, Nanjing Medical University, Nanjing, Jiangsu, 210029, China; Department of Medical Informatics, School of Biomedical Engineering and Informatics, Nanjing Medical University, Nanjing, Jiangsu, 210009, China; Department of Medical Informatics, School of Biomedical Engineering and Informatics, Nanjing Medical University, Nanjing, Jiangsu, 210009, China; Department of Information, The First Affiliated Hospital, Nanjing Medical University, Nanjing, Jiangsu, 210029, China

**Keywords:** EHR pre-trained model, type 2 diabetes mellitus, depression and anxiety, regional EHRs, deep learning

## Abstract

**Objective:**

Due to heterogeneity and limited medical data in primary healthcare services (PHS), assessing the psychological risk of type 2 diabetes mellitus (T2DM) patients in PHS is difficult. Using unsupervised contrastive pre-training, we proposed a deep learning framework named depression and anxiety prediction (DAP) to predict depression and anxiety in T2DM patients.

**Materials and Methods:**

The DAP model consists of two sub-models. Firstly, the pre-trained model of DAP used unlabeled discharge records of 85 085 T2DM patients from the First Affiliated Hospital of Nanjing Medical University for unsupervised contrastive learning on heterogeneous electronic health records (EHRs). Secondly, the fine-tuned model of DAP used case–control cohorts (17 491 patients) selected from 149 596 T2DM patients’ EHRs in the Nanjing Health Information Platform (NHIP). The DAP model was validated in 1028 patients from PHS in NHIP. Evaluation included receiver operating characteristic area under the curve (ROC-AUC) and precision-recall area under the curve (PR-AUC), and decision curve analysis (DCA).

**Results:**

The pre-training step allowed the DAP model to converge at a faster rate. The fine-tuned DAP model significantly outperformed the baseline models (logistic regression, extreme gradient boosting, and random forest) with ROC-AUC of 0.91±0.028 and PR-AUC of 0.80±0.067 in 10-fold internal validation, and with ROC-AUC of 0.75 ± 0.045 and PR-AUC of 0.47 ± 0.081 in external validation. The DCA indicate the clinical potential of the DAP model.

**Conclusion:**

The DAP model effectively predicted post-discharge depression and anxiety in T2DM patients from PHS, reducing data fragmentation and limitations. This study highlights the DAP model’s potential for early detection and intervention in depression and anxiety, improving outcomes for diabetes patients.

## Introduction

The prevalence of depression and anxiety in patients with diabetes is twice that of the non-diabetic population.^1^ These psychological disorders affect the self-management, glycemic control adversely,^2^ and are associated with increased risk of cardiovascular complications and dementia,^3–5^ more healthcare resources consumption,^6^ higher healthcare costs,^7^^,^^8^ and increased risk of all-cause hospitalization for patients with diabetes.^9^ Annual mean total healthcare costs were higher for diabetes patients with comorbid depression (EUR 5629 [95% CI 4987-6407]) than without (EUR 3252 [95% CI 2976-3675]).^10^ Predicting depression or anxiety in patients with diabetes is critical for optimizing glycemic control and reducing the cost.

Primary healthcare services (PHS) are crucial for meeting the needs of alleviating the burden of the disease, improving diabetes management,^11^ and also the primary sources of mental healthcare.^12^ The recent proposals outlined in the “Healthy China 2030” suggested that the physicians of PHS in China will need to become a pivotal role for mental health therapies in the future.^13^ Therefore, PHS are expected to become an important mental health identification approach for patients with diabetes.^14^

The commonly used methods for assessing depression and anxiety present practical challenges in PHS. To assess the severity of depression or anxiety in diabetic patients, it primarily relies on the total symptom scores reported by screening tools, including Geriatric Depression Scale (GDS),^15^^,^^16^ the Patient Health Questionnaire-9 (PHQ-9) scale,^17^ Generalized Anxiety Disorder Scale-7 (GAD-7),^17^ and etc. However, the aforementioned tools are prone to fluctuations in symptom perception and can be influenced by recent events.^18^ Primary healthcare services often lack the involvement of mental health specialists. This has resulted in a delay in alerting to the risk of depression in diabetic patients and has consequently impacted early intervention efforts.

Electronic health records (EHRs) contain demographic information, symptoms, medical treatment processes, medication, medical history, images, laboratory tests, and other data from patients’ previous follow-up visits. In addition, patients’ medical history and reported symptoms are documented in an unstructured form within free-text notes. However, despite the existence of numerous models identifying the risk of depression or anxiety by leveraging patients’ EHRs,^19–21^ there are two main reasons that hinder the identification of depression and anxiety among diabetes patients in PHS.

Firstly, existing models struggle to comprehensively capture the heterogeneous nature of EHR data when exclusively utilizing structured information, leading to a decrease in their effectiveness. The majority of current EHR-based models for predicting mental health rely on structured data with extracted topics^20^ or symptoms^21^ from text, and necessitate manual conversion of unstructured data into structured formats before training. However, the performances of existing prediction models^19–21^ are far from satisfactory. Our hypothesis is that the step of defining on what to be extracted from free-text limits the ability of data driven approaches (eg, deep learning models) for identifying unknown associations with depression/anxiety complications.

Furthermore, there is a two-faceted technical challenge of deriving models directly from Chinese PHS settings. First, in China, the PHS is significantly underused^22^ compared to those in the United States and the United Kingdom. This leads to the fact that data from primary care is not as widely available. Second, primary care settings have limited personnel qualified to conduct psychotherapy and antidepressants standardly unavailable. Patients can only receive psychological diagnosis or mental health services through referrals.^23^ The two combined lead to scarcity and fragmentation of depression-related diagnostic data for PHS patients. Consequently, these obstacles hinder the performance of (directly PHS derived) predictive models for depression in patients with diabetes.^24^

Large-scale labeled data on EHRs is scarce, while EHR data itself is huge in volume. Recently, contrastive learning, making the model associate similar and dissociate dissimilar samples, is becoming a major form of self-supervised pre-training in the first phase.^25^ By using large unlabeled datasets to pre-train machine-learning models, self-supervised learning improves the performance of downstream tasks.^26^ Models with contrastive self-supervised pre-training have required fewer labelled examples to reach the same performance than models trained only through supervised learning.^26^ Through fine-tuning in next phase, the pre-trained model can be applied to various specific tasks related to diabetes patients.

This study addressed the aforementioned challenges of predicting depression and anxiety in patients with diabetes by developing an EHR-based model called the Depression and Anxiety Prediction (DAP) model. The model utilized an unsupervised pre-training approach using discharge records from T2DM patients in multiple healthcare services and validated it in PHS. This study highlights the effectiveness of the DAP model in early detection and intervention for mental health conditions in diabetes patients, contributing to improved healthcare outcomes.

## Materials and methods

**Figure 1.**
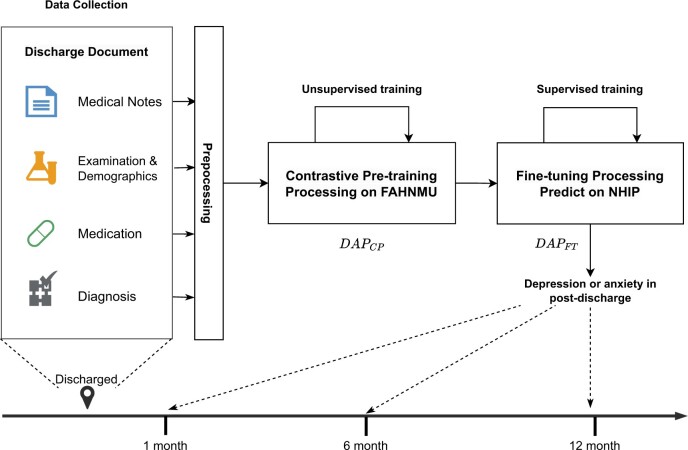
The paradigm of the depression and anxiety prediction (DAP) model.

Our study was conducted in two steps (Figure 1), inspired by clinical event forecasting model.^25^ In the first step, we collected and processed the hospital records of T2DM patients from the First Affiliated Hospital of Nanjing Medical University (FAHNMU). Using unsupervised learning, we developed a contrastive pre-training model for EHRs on this dataset. In the second step, we constructed cohorts for the occurrence of depression or anxiety in post-discharged patients with T2DM from the Nanjing Health Information Platform (NHIP). Then, we fine-tuned our EHR pre-trained model to predict the risk of depression or anxiety in T2DM patients during multiple periods after discharge. This approach has been used before^27^^,^^28^ and enables modeling of changes in risk over time (new predicted risk for each included admission) within patients. Ethical approval (2020-SR-163) for the study was received from the Ethics Committee of the First Affiliated Hospital, Nanjing Medical University, Jiangsu, China.

### Data collection and cohorts construction

#### Discharged EHR dataset of T2DM patients from FAHNMU

First, we constructed a discharged EHR dataset for T2DM diabetic patients (169 058 participants) who visited the FAHNMU between January 1, 2016 and October 31, 2022. Patients with diabetes were included if they met the following screening criteria: hemoglobin A1C (HbA1c) ≥ 48 mmol/mol, or 6.5%, or use of anti-diabetic medications, or presence of a diabetes diagnosis or medical history. No exclusion criteria based on age or diagnosed disorder were applied. To ensure the use of high-quality data during the pre-training process, descriptions unrelated to the disease or symptoms (eg, physical examination, consultation, medication, etc) and non-informative chief complaint descriptions (eg, unclear, unknown, etc) were removed. This produced a total of 183 662 electronic records from 85 085 patients in the database used for pre-training process.

Each discharge document comprised structured and unstructured data. The structured data comprised demographic information (age, sex, marital status) and laboratory values. The general demographics of one patient in different hospitalizations were treated as different samples following.^28^ Outlier detection was performed on numeric values using the IsolationForest algorithm,^29^ followed by normalization and division into discrete intervals. The unstructured data consisted of diagnosis labels and medical notes containing patient complaints, history of the present illness, past illnesses, and family medical history. To prepare the data for the unsupervised pre-training process, we aggregated general demographics, diagnoses of discharge, laboratory values during the hospitalization, and notes of medical history for each hospitalization.

#### Case–control cohorts construction from NHIP

Nanjing Health Information Platform is an integrated medical information platform containing the EHRs from most healthcare services in Nanjing, Jiangsu, China. Inpatients from NHIP who had already been diagnosed with diabetes and discharged between January 1, 2020 and December 31, 2021 were selected. To avoid data leaking caused by the pre-training process, the discharge EHRs from FAHNMU and PHS were excluded. The discharge EHR dataset for T2DM patients in NHIP was constructed the same as section discharged EHR dataset of T2DM patients from FAHNMU.

The case cohort was defined as the discharge records of T2DM patients from NHIP who experienced depression or anxiety events within one year after discharge. Depression or anxiety events refer to the presence of depression or anxiety diagnoses or the use of antidepressant or anxiolytic medications. Depression refers to diagnoses with the International Classification of Diseases (ICD)-10 codes F31-F34, F39, F06.3, or the use of antidepressant medications mentioned in the Anatomical Therapeutic Chemical (ATC) code N06A (https://www.whocc.no/atc_ddd_index/?code=N06A). Anxiety refers to diagnoses with ICD-10 codes F40-F43, F06.4, or the use of anxiolytics with the ATC code N05B (https://www.whocc.no/atc_ddd_index/?code=N05B). To avoid the influence of short-term depression or anxiety in patients, we excluded hospitalizations that had occurrences of depression or anxiety within six months prior to discharge diagnosis. Additionally, we merged repeated EHR records of T2DM patients within one week and selected the latest record.

For the control-cohort, we followed the propensity score matching (PSM) approach from Lau Raket et al^30^ by selecting hospitalizations with similar discharge times and similar outcome event times (in this case, number of days between discharge dates and last date within the time window). We constructed datasets with case–control record ratios of 1:3 to simulate the rate of depression in patients with T2DM (25%^31^^,^^32^) (Appendix S2, Figure SA1). Similarly, the process of constructing case–control cohorts for depression or anxiety prediction tasks within 30 and 180 days after discharge followed the same procedure.

Primary healthcare services are essential for the management and support of diabetic patients. Therefore, to validate the performance of our model in such services, we selected 78 PHS (including community medical service centers, community hospitals, etc) from NHIP as sub-cohorts and constructed a case–control sub-cohort using the same PSM method. Also, we selected a large general healthcare service (${\text{GHS}}_{L}$), a medium general healthcare service (${\text{GHS}}_{M}$), and a Traditional Chinese Medicine healthcare service (TCM-HS). These sub-cohorts (1:3) were constructed using the same PSM method.

### DAP model development

#### Pre-training process on discharged EHR dataset of T2DM patients

**Figure 2.**
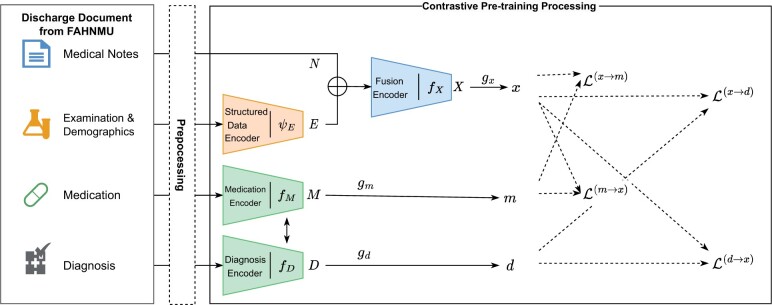
The structure of the $DAP_{CP}$ model. The *x*, *m*, *d* were the outputs of $DAP_{CP}$ model as one encoded discharge document. The dotted part with arrow is the calculation path of the loss function.

We established a contrastive pre-training model ($DAP_{CP}$, Figure 2) based on discharged EHR dataset from section Discharged EHR dataset of T2DM patients from FAHNMU. The objective of the model was to minimize the internal distance between inpatient medical text, personal information, laboratory test data, and discharge diagnosis in each record via contrastive learning. We constructed separate encoders for unstructured and structured data due to the heterogeneous nature of EHR data.

For structured data, specifically demographic information and laboratory test results within a single record, we concatenated embedding of discretized data and the representation of feature names from a large-scale language model (LLM)^33^ before feeding it into the structured encoder $\psi_{E}$, which was a general transformer structure.^34^ For unstructured textual data, such as medical history, diagnosis names, medication names, and laboratory test names, we first utilized LLM for static encoding to obtain their representation vectors as inputs to our model. This step can be considered as part of our data pre-processing. For the patient’s medical history, diagnostic text, and medication record text were encoded using text encoders with the same structure, denoted as $f_{X}$, $f_{D}$, and $f_{M}$, respectively.

In order to fusion the unstructured and structured of data (the patient’s medical history and the laboratory test results during hospitalization), we applied Fast Linear Attention with a Single Head (FLASH) model as the fusion encoding model, denoted as $f_{X}$ that can support a length of over 2500.^35^ The result *E* from $\psi_{E}$ was concatenated with an encoded medical record *N* from LLM as input. The algorithmic process of the fusion function $f_{X}$ is illustrated in Algorithm 1, where *W* denotes the learn-able variables, and *S* denotes the length of the input.

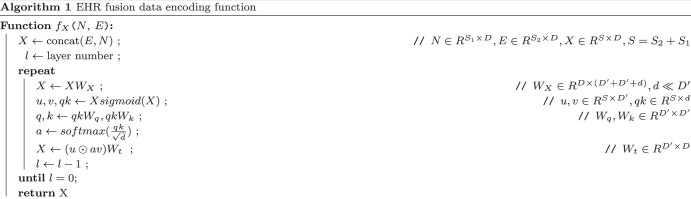

We hypothesized a potential connection between a patient’s medical history, laboratory test data, and the diagnoses and medications prescribed by doctors, based on common sense. To model this connection, we employed contrastive learning—a discriminative technique that enhances semantic similarity among predefined instances within the same class while reducing semantic similarity between different instances.^36^ The objective loss function is defined as eqn (1) where τ is a temperature hyper-parameter, and *q* is similar to its positive key $k_{+}$ and dissimilar to all other keys considered negative keys for *q* in one batch *K*.

(1) $$L^{\left(q,k\right)}=-\text{log}\;\frac{\;\text{exp}\;\left(q\cdot k_{+}/\tau \right)}{\sum_{i=0}^{K}\;\text{exp}\;\left(q\cdot k_{i}/\tau \right)}$$

We raised a paired contrastive loss L (eqn (2)) to enhance the latent relation between the patient’s information representation and the representation of diagnosis or medications, which was inspired by the contrastive loss function of the contrastive language-image pre-training (CLIP).^37^ The outputs *x*, *m*, *d* were get via three fully connected networks $g_{x}$, $g_{m}$, and $g_{d}$ which accepted *X*, *M*, *D* as inputs. Then, we optimized the *x* similarity with corresponding discharge diagnoses *d* and medication *m* through the $L_{CP}$.

(2) $$L_{CP}=L^{\left(x,m\right)}+L^{\left(m,x\right)}+L^{\left(x,d\right)}+L^{\left(d,x\right)}$$

#### Fine-tuning process for depression and anxiety prediction

To predict the occurrence of depression or anxiety within one year after discharge in T2DM patients from case–control cohorts, we conducted a fine-tuning model ($DAP_{FT}$) on the $DAP_{CP}$ model obtained from section pre-training process on discharged EHR dataset of T2DM patients. Our optimization objective function ($L_{FT}$, eqn (3), where *y* represents the ground truth label and *u* represents the predicted probability) was to perform prediction tasks on multiple time intervals after discharge. Firstly, we encoded EHR data from case–control cohorts using the pre-trained model, obtaining the latent representations *x*, *m*, *d* of each EHR record. Then, *x*, *m*, *d* were concentrated and inputted into a single-layer neural network as $\phi_{U}$ to classify whether depression or anxiety existed in the following time intervals.

(3) $$L_{FT}=\sum_{i}\;-\;\left[y_{i}\cdot \;\text{log}\;\left(u_{i}\right)+\left(1\;-\;y_{i}\right)\cdot \;\text{log}\;\left(1\;-\;u_{i}\right)\right]$$

### Evaluations and interpretation of models

The demographic characteristics of the records in the case–control cohorts were compared and tested for potential differences at a significance level of 0.05. Categorical variables were analyzed using $\chi^{2}$ tests, while continuous variables were evaluated using Wilcoxon tests, all for descriptive purposes.

We employed machine learning models, such as logistic regression (LR), extreme gradient boosting (XGB), and random forest (RF), as baseline models to compare the performance of DAP model. The LR, XGB, and RF had been selected as comparator baseline models in various researches and proved effectiveness in predicting depression on EHRs.^38–41^ Missing values in the structured data were filled using mean imputation, while the unstructured data were vectorized using the term frequency-inverse document frequency (TF-IDF).^42^ The two types of vectors for each record were concatenated and input into the baseline models. Additionally, we also validated the Latent Dirichlet allocation (LDA) method as vectorization of unstructured data^43^ (see Appendix Tables SA6, SA7). In the overall NHIP cohort, after excluding PHS and FAHNMU, the $DAP_{FT}$ model was subjected to 10-fold cross-validation on both NHIP and PHS. Additionally, NHIP and three sub-cohorts (${\text{GHS}}_{L}$, ${\text{GHS}}_{M}$, TCM-HS) were used as training data for external validation on PHS.

The mean value of receiver operating characteristic area under the curve (ROC-AUC) and precision-recall area under the curve (PR-AUC) were evaluated as metrics. The *t*-test is used to compare the differences between two groups of indicators (10-fold ROC-AUC and PR-AUC). Furthermore, decision curve analyses (DCAs)^44^ were utilized to assess the clinical utility of various prediction models by considering the balance between the benefits and harms associated with different decision thresholds. During DCA, the net benefit of each model is evaluated across a spectrum of threshold probabilities that reflect the probability at which a clinician or patient would take action based on the prediction. The model that provides the greatest net benefit over the complete range of threshold probabilities is deemed to have the highest clinical utility. In the DCA figure, the solid lines and shaded areas correspond to the means and standard deviations of the net benefit of each model in 10-fold validation.

To interpret the DAP model and find the most influential features for depression and anxiety prediction, we applied the integrated gradients (IG) method^45^ using the Python Captum library^46^ released by Facebook to calculate the mean attribute scores of input features. The idea behind IG is to compute the gradients of the model’s prediction with respect to the input features while integrating these gradients along a path from a reference input $x^{\prime }$ to the actual input *x*. The IG score along the $i^{th}$ dimension for an input *x* is defined in eqn (4), where α is the scaling coefficient, F(x) represents our DAP model, and $\frac{\partial F\left(x\right)}{\partial x_{i}}$ is the gradient of DAP model F(x) along the $i^{th}$ dimension. To calculate the IG score for each feature, we summed up all the dimensions included in each feature (diagnosis, laboratory tests, medications). This produced the IG score for each feature, and we listed the top 20 feature names.

(4) $$IG_{i}(x)::=\left(x_{i}\;-\;x_{i}^{\prime }\right)\times \int_{\alpha =0}^{1}\frac{\partial F\left(x^{\prime }+\alpha \times \left(x\;-\;x^{\prime }\right)\right)}{\partial x_{i}}d\alpha$$

### Implementation details

The PSM method for constructing the cohorts was performed using the PsmPy package provided by Owens-Gary et al.^47^ The deep learning model was implemented in PyTorch version 1.11.0. Max length of text input was 2500, layer number was 24, batch size was 6, epoch length was 10, and learning rate was 2e−5. We used 4 NVIDIA GeForce RTX 3090 GPU of 24GB graphics memory capacity. Machine learning models were implemented in scikit-learn package version 1.0.2 and Pycare 3.0. We used the default hyper-parameters for each model.

## Results

### Descriptive results of FAHNMU and NHIP

We employed a cohort of 85 085 hospitalized patients (183 662 discharge records) from FAHNMU to construct the $DAP_{CP}$ model for EHR (Appendix S1, Table SA1). Among these patients, males accounted for 58.94%, and the median age was 65 years. Notably, 5.76% of patients experienced depressive or anxious episodes within one year after discharge, with a median duration of 91 days post-discharge (IQR: 173.42). In the NHIP cohort, after excluding hospitalization records from FAHNMU, a total of 149 596 hospitalized patients (251 361 discharge records) remained. Males constituted 56.56% of this cohort, and the median age mirrored that of FAHNMU. Remarkably, 28.21 cases of depression or anxiety occurred for every 1000 person-year in NHIP, which was twice compared with FAHNMU. As for PHS, the prevalence of depression or anxiety was 22.11 for every 1000 person-year.

We derived five pairs of case–control cohorts from the discharge records of T2DM patients in NHIP, focusing on the occurrence of depression or anxiety within three specific post-discharge time intervals. Taking 365 days post discharge as an example (Table 1), discharge records indicating depression or anxiety within this period were classified into the case cohort. In NHIP, the case cohort contained 5445 discharge records and exhibited a higher proportion of females (51.69%) and older age of admission (median age: 68 years), in contrast to the gender and age distribution observed in the control cohort. As in PHS, 286 records showed occurrence of depression or anxiety within 365 days post discharge. The data description tables for ${\text{GHS}}_{M}$ and ${\text{GHS}}_{L}$ can be found in Table SA2 of Appendix S1.

**Table 1.** Descriptive result of the case–control cohorts from NHIP and PHS.

	NHIP^a^			PHS		
	Case (*n* = 5445)	Control (*n* = 16 335)	*P*-value	Case (*n *= 286)	Control (*n* = 858)	*P*-value
Patients, *N*	3678	13 813		203	825	
Gender, *N* (%)						
Female	1901 (51.69)	5764 (41.73)	.000	119 (58.62)	437 (52.97)	.171
Male	1777 (48.31)	8049 (58.27)		84 (41.38)	388 (47.03)	
Age, year (IQR)	68 (17)	66 (18)	.000	69 (12)	70 (12)	.814
Examination, median (IQR)						
Temperature, °C	36.50 (0.30)	36.50 (0.30)	.072	36.50 (0.40)	36.50 (0.30)	.110
Pulse, times	78.00 (14.00)	78.00 (14.00)	.000	76.00 (15.00)	76.00 (14.00)	.695
SBP, mm Hg	133.00 (29.00)	133.00 (26.00)	.277	133.00 (27.00)	138.00 (21.00)	.001
DBP, mm Hg	79.00 (16.00)	80.00 (16.00)	.061	80.00 (13.00)	80.00 (13.00)	.812
GLU, mmol/L	6.33 (3.08)	6.84 (3.51)	.000	7.23 (3.24)	7.95 (4.02)	.341
HbA1c, %	6.90 (1.90)	7.30 (2.40)	.000	7.50 (2.20)	7.35 (2.62)	.839

a The records in PHS were removed.

Abbreviations: DBP, diastolic blood pressure; GLU, glucose; HbA1c, glycosylated hemoglobin; NHIP, Nanjing Health Information Platform; PHS, primary healthcare services; SBP, systolic blood pressure.

### Performance of fine-tuning model for depression and anxiety

#### Internal validation for NHIP and PHS

**Figure 3.**
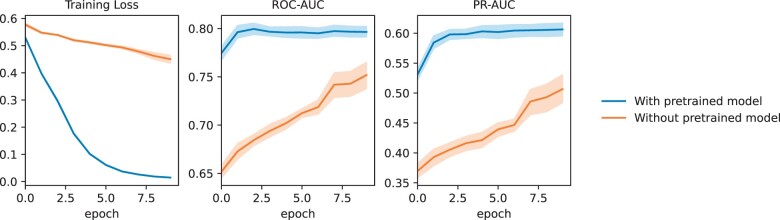
During the training process of the depression and anxiety prediction (DAP) model within 365 days on the Nanjing Health Information Platform (NHIP) case–control cohorts, the changes of various indicators on 10-folder validation were compared between the use of pre-training models and the absence of pre-training models. The metrics include training loss, ROC-AUC, and PR-AUC on the each fold of test dataset.

We conducted an ablation experiment to compare the performance of the DAP model without pre-training and the DAP on the NHIP dataset, as shown in Figure 3, with 10-fold internal validation results for each epoch. The DAP model achieved stable ROC-AUC and PR-AUC performance as early as the second epoch. This suggested that we do not actually need to train for 10 epochs to achieve optimal performance, thus saving computational resources.

We compared our fine-tuned pre-trained model with baseline models (including XGB, RF, and LR) to evaluate the performance in predicting the occurrence of depression or anxiety risk within 365 days of the post-discharge time interval. In the 10-fold internal validation on the PHS, as shown in Table 2, the DAP model significantly outperformed the baseline models (ROC-AUC: 0.91 ± 0.028, PR-AUC: 0.80 ± 0.067, *P*-value<.000). Furthermore, it also demonstrated excellent predictive performance on the overall NHIP dataset. For the tasks of predicting depression and anxiety at 30 days and 180 days after discharge, performance metrics can be found in the tables in Appendix S2. The predictive performance at 30 days was not significantly different from baselines.

**Table 2.** Performance of DAP model and baseline models within 365 days after discharge on 10-fold internal validation for cohorts from NHIP and PHS.

Models	ROC-AUC	*P*-value	PR-AUC	*P*-value
NHIP^a^				
LR	0.60 (±0.011)	.000	0.32 (±0.009)	.000
RF	0.73 (±0.011)	.000	0.51 (±0.015)	.000
XGB	0.72 (±0.012)	.000	0.46 (±0.026)	.000
DAP	**0.80** (±0.010)	ref	**0.61** (±0.018)	ref
PHS				
LR	0.60 (±0.072)	.000	0.41 (±0.101)	.000
RF	0.60 (±0.057)	.000	0.34 (±0.056)	.000
XGB	0.65 (±0.072)	.000	0.41 (±0.072)	.000
DAP	**0.91** (±0.028)	ref	**0.80** (±0.067)	ref

a The records from PHS were removed.

The numbers in parentheses are the standard deviation. The bolded part indicates the best performance of the corresponding data under the respective metric.

Evaluation metrics included ROC-AUC and PR-AUC. We conducted a *t*-test to compare the differences in results between the two groups generated from the 10-fold data.

Abbreviations: DAP, depression and anxiety prediction; LR, logistic regression; NHIP, Nanjing Health Information Platform; PHS, primary healthcare services; RF, random forest; XGB, extreme gradient boosting.

#### External validation on PHS for sub-cohorts

We used PHS as validation data for 10-folds validations to compare the predictive performance using different types of healthcare service institution data. As shown in Table 3, using NHIP (excluding records from PHS) as the training data, we validated the performance of the DAP model in predicting the occurrence of depression and anxiety after 365 days of discharge for diabetic patients on PHS. The DAP model achieved significant advantages (ROC–AUC: 0.75±0.045, *P*<.000; PR-AUC: 0.47±0.081, *P*<.000). Among the sub-cohorts of the three types of healthcare services, the DAP model demonstrated significant superiority over the baselines in the TCM-HS cohorts (ROC-AUC: 0.74±0.035, *P*<.000; PR-AUC: 0.46±0.073, *P*<.000). However, the DAP model did not show significant performance advantages on GHS data, regardless of the scale of the general healthcare service (GHS) data. For the tasks of predicting depression and anxiety at 30 days and 180 days after discharge, performance metrics can be found in the tables in Appendix S2.

**Table 3.** Performance of DAP model and baseline models within 365 days after discharge on 10-fold external validation on PHS for cohorts from NHIP, GHS, and TCM-HS.

Models	ROC-AUC	*P*-value	PR-AUC	*P*-value
NHIP^a^				
LR	0.51 (±0.076)	.000	0.30 (±0.074)	.000
RF	0.52 (±0.061)	.000	0.28 (±0.058)	.000
XGB	0.53 (±0.065)	.000	0.26 (±0.039)	.000
DAP	**0.75** (±0.045)	ref	**0.47** (±0.081)	ref
GHS_L_				
LR	0.50 (±0.055)	.000	0.28 (±0.060)	.009
RF	0.50 (±0.061)	.000	0.27 (±0.037)	.000
XGB	0.61 (±0.051)	.476	**0.37** (±0.053)	.804
DAP	**0.62** (±0.059)	ref	0.36 (±0.055)	ref
GHS_M_				
LR	0.40 (±0.069)	.005	0.23 (±0.042)	.281
RF	**0.50** (±0.059)	.737	0.27 (±0.050)	.453
XGB	0.49 (±0.063)	.848	**0.27** (±0.037)	.473
DAP	0.49 (±0.060)	ref	0.26 (±0.053)	ref
TCM-HS				
LR	0.56 (±0.058)	.000	0.33 (±0.053)	.000
RF	0.55 (±0.056)	.000	0.30 (±0.036)	.000
XGB	0.56 (±0.066)	.000	0.31 (±0.066)	.000
DAP	**0.74** (±0.035)	ref	**0.46** (±0.073)	ref

a The records from PHS were removed.

The numbers in parentheses are the standard deviation. The bolded part indicates the best performance of the corresponding data under the respective metric. Evaluation metrics included ROC-AUC and PR-AUC. We conducted a *t*-test to compare the differences in results between the two groups generated from the 10-fold data.

Abbreviations: DAP, depression and anxiety prediction; LR, logistic regression; NHIP, Nanjing Health Information Platform; PHS, primary healthcare services; RF, random forest; TCM-HS, Traditional Chinese Medicine healthcare service; XGB, extreme gradient boosting.

**Figure 4.**
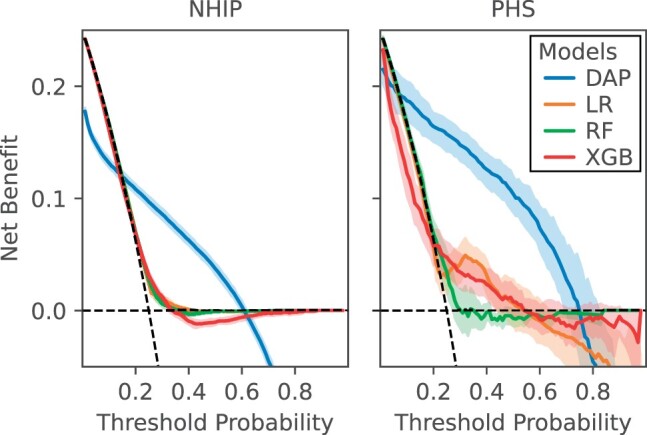
Comparison of decision curve analysis between depression and anxiety prediction (DAP) model and baseline models (logistic regression [LR], random forest [RF], extreme gradient boosting [XGB]) for depression or anxiety risk prediction in 365 days for cohorts from Nanjing Health Information Platform (NHIP) and primary healthcare services (PHS). The net benefit of the decision curve analysis (DCA) curve is calculated based on the 10-fold internal validation.

**Figure 5.**
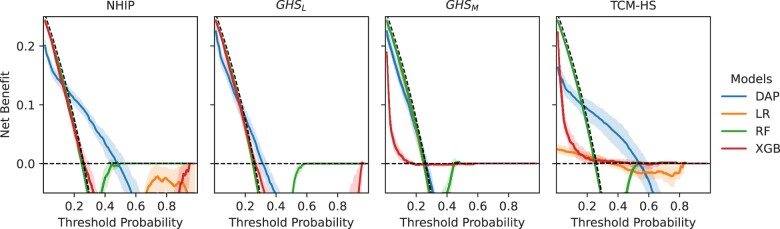
Comparison of decision curve analysis between depression and anxiety prediction (DAP) model and baseline models (logistic regression [LR], random forest [RF], extreme gradient boosting [XGB]) for depression or anxiety risk prediction in 365 days. The net benefit of the decision curve analysis (DCA) curve is calculated based on the 10-fold validation on the primary healthcare services (PHS).

The DCA curves illustrated that the net benefit of the DAP model trained on NHIP, TCM-HS, and PHS surpassed that of the baseline models within the threshold range of 0.3 to 0.5 in 10-fold validation (Figures 4 and 5). This observation suggested that the fine-tuned pre-trained model was more adept at striking a balance between accurately identifying true positives and minimizing false positives.

### Interpretation of DAP model

To explain the model’s prediction for depression and anxiety, we summed up the input features across dimensions and obtained the IG score for each feature. The features included diagnosis, medication names, laboratory tests, and patient disease information. The diagnosis adopted the ICD-10 coding system. For medications, we removed the descriptions of dosage forms to merge similar medications.

The top 20 mean IG scores of the feature texts from captum are shown in Table 4. The top three feature items with the highest positive predictive contribution for depression or anxiety are postherpetic neuralgia diagnosis, finasteride medication, and burn corrosion.

**Table 4.** Based on the top 20 feature attribution scores provided by Captum, a detail column is presented, indicating the corresponding ICD codes in relation to the textual content of the original discharge diagnosis.

Feature name	Type	IG Score
Sequelae of other and unspecified infectious and parasitic diseases, B94	Diagnosis	4.09
Finasteride	Drug	4.04
Burn and corrosion, body region unspecified, T30	Diagnosis	3.02
Malignant neoplasm of uterus, part unspecified, C56	Diagnosis	2.98
Telmisartan	Drug	2.69
Malaise and fatigue, R53	Diagnosis	2.52
Other disorders of pancreatic internal secretion, E16	Diagnosis	1.85
Malignant neoplasm of vulva, C51	Diagnosis	1.83
Other inflammatory liver diseases, K75	Diagnosis	1.81
Abnormal results of function studies, R94	Diagnosis	1.77
Omeprazole sodium	Drug	1.765
Acute myocardial infarction, I21	Diagnosis	1.70
Subarachnoid hemorrhage, I60	Diagnosis	1.67
Recombinant lysine-protein zinc insulin	Drug	1.67
Propranolol hydrochloride	Drug	1.67
Nao Xin Qing	Drug	1.63
Recombinant human insulin zinc	Drug	1.57
Edaravone	Drug	1.52
Nonorganic sleep disorders, F51	Diagnosis	1.51

## Discussion

In this study, we constructed the DAP model to provide risk prediction for the occurrence of depression or anxiety in multiple time periods for patients with T2DM, and addressed the issues of data heterogeneity and scarcity in the records from PHS. The DAP model is followed by two phases of training (self-supervised pre-training called $DAP_{CP}$ and supervised fine-tuning called $DAP_{FT}$) on EHRs. Through validation on a large NHIP cohort and a small PHS cohort, DAP model demonstrated significantly superior ROC-AUC and PR-AUC metrics compared to the baseline models. Furthermore, to explore the clinical utility of our model, we conducted DCA, revealing its stable advantage at specific thresholds on the PHS.

The DAP model can assist PHS in identifying the risks of depression or anxiety in T2DM patients via EHR. In recent years, artificial intelligence algorithms have been widely applied in the field of depression or anxiety prediction. Previous researches have mostly been based on scale tools^48^ or structured data from EHR.^20^^,^^49^ Gettings et al^48^ developed Patient Health Questionnaire for Adolescents (PHQ-A) to increase the sensitivity of depression screening for youth with diabetes. Hochman et al^20^ used XGB model predicting postpartum depression among women by analyzing demographics, medication prescriptions, laboratory measurements, and other EHR data. Song et al^49^ identified eight bio-markers from EHR to predict depression in diabetes mellitus using support vector machine. However, PHS lack specialized training in providing psychological health services.^24^ In addition, scarcity of features in trainable data and low data completeness in PHS could have obstacles in prediction depression or anxiety in patients with diabetes. The DAP model achieved the best performance (0.91±0.028 of ROC-AUC and 0.80±0.067 of PR-AUC) compared to the XGB model, when using PHS data only. Also, it is worth noting that although TCM-HS have less than 20% of the NHIP training data, the performance in validating the PHS (ROC: 0.74±0.035) is close to that of using the NHIP data (ROC: 0.75±0.045). The annual incidence rate of depression among inpatients in TCM-HS is 29 per thousand persons, which is close to the rate in NHIP of 28.21, indicating that it is unrelated to the occurrence of depression. We speculate that this may be related to the content of medical record text written by doctors in TCM-HS, which requires further analysis using natural language processing tools in the future.

The initial component of the DAP model, $DAP_{CP}$ model, effectively captured the heterogeneity of EHR data, including textual data, structured laboratory tests, and demographic information. This $DAP_{CP}$ is based on long-term and high-quality FAHNMU data, serving as the foundation to the challenges predicting depression and anxiety among diabetes patients using records from PHS. In contrast to Zhang et al’s approach,^25^ which employed contrastive learning on temporally close medical records for the same patient, we do not follow this method due to the long time span and potential dissimilarities within our inpatient cohort for T2DM in our data. Consequently, we validated our hypothesis that there exists inherent similarity among various sections (eg, medical history, examinations, medications, and diagnoses) within discharge documents. By employing our $DAP_{CP}$ pre-training model and subsequently fine-tuning it on NHIP data, we have observed a significant enhancement in the prediction model for depression and anxiety. This improvement is particularly evident when compared to models without pre-training, as our approach demonstrates faster convergence. These findings affirm the reliability of our pre-training model construction method and highlight its potential for future fine-tuning tasks targeting other diseases in T2DM patients.

We used the IG score to demonstrate the factors that contribute to the prediction of depression and anxiety in patients with diabetes. We calculate the IG score for each feature by summing the input features across dimensions. Laboratory tests have a relatively low impact on predicting the occurrence of depression and anxiety after discharge in T2DM patients. The main influential features are derived from patients’ discharge diagnosis and medication. We found that herpes zoster, burn, and finasteride are the top three features with the highest IG scores, and these three factors have been reported related to the occurrence of depression.^50–52^ Additionally, we found that telmisartan is also an important feature for predicting the occurrence of depression or anxiety. Telmisartan can induce central angiotensin type 1 receptor blockade and has the potential to be an oral antidepressant.^53^ However, studies have also shown that high doses of telmisartan can induce depressive symptoms in diabetes-induced depression rat.^54^ Therefore, the antidepressant effect of this medication still need further discussion in the future.

Our study has the following limitations. First, the DAP model directly uses diagnostic text for contrastive learning in the pre-training process of comparing discharge documents, which may lack the correlation between different diagnoses. For example, diabetes and thyroiditis both belong to endocrine system diseases. In the future, this limitation could be addressed by adding hierarchical structure task prediction between diagnoses to establish associations. Second, there is a possibility of false positives among the control group in each cohort, which is also a common issue in many prediction models related to mental health. When constructing the case–control training data, for the control group of negative T2DM patients, the determination is based on the presence of medical records and the absence of depression or anxiety events. In the future, we will enhance and improve our model by integrating with the medical data platform of Jiangsu Province. Third, the inclusion of hospitalized patients could introduce a potential source of bias, and patients may differ in significant ways from those who do not require hospitalization. In future research, we could explore these differences in more detail and strength our model’s generalizability. Finally, the performance of predicting the occurrence risk of depression or anxiety after discharge for T2DM patients should be confirmed through prospective clinical experiments. Previous studies have conducted prospective clinical validation of prediction models for short-term mental health crises after healthcare visits^40^ and affirmed their clinical value. Diabetes is a chronic disease, so it is crucial to predict the long-term mental status of diabetic patients and conduct prospective validation. Additional research is needed in the future to not only evaluate the model’s performance but also assess whether it can provide benefits for glycemic management.

## Conclusion

Overall, our study validates the feasibility of constructing a T2DM patient EHR contrastive pre-training model on FAHNMU and using it to fine-tune the risk of depression or anxiety in discharged patients across multiple time periods in regional EHR data, especially in PHS. Through data validation across multiple institutions, the model has demonstrated potential for clinical applications. Population-based validation and addressing challenges that may arise in clinical practice should be included in future considerations.

## Supplementary Material

ocad228_Supplementary_Data

## Data Availability

The data underlying this article cannot be shared publicly due to the potential identifying nature of the records. The data will be shared on reasonable request to the corresponding author. The code of this study is available in https://github.com/inseptember/DAP.
