# Supplementary material for: Applying contrastive pre-training for depression and anxiety risk prediction in type 2 diabetes patients based on heterogeneous electronic health records: a primary healthcare case study
Source: J Am Med Inform Assoc. 2023 Dec 7;31(2):445–55. doi: 10.1093/jamia/ocad228 (PMC10797279; doi:10.1093/jamia/ocad228)
Supplement: ocad228_Supplementary_Data [file ocad228_supplementary_data.zip › ocad228_Supplementary_Data/Appendice_1.pdf]

Table A1: Description of the T2DM patients discharge EHRs from JSPH, and the regional NHIP.

	JSPH // (n=183662)		NHIP // (n=251361)		p-value
Total patients, N	85085		149596		
Gender					
Female, N (%)	34938 (41.06)		64983 (43.44)		0.000
Male, N (%)	50147 (58.94)		84613 (56.56)		
Age, Year (IQR)	65 (18)		65 (17)		0.000
Diabetes Duration, Year (IQR)	0.28 (4.21)		4.46 (4.95)		0.000
Depression or Anxiety, N (%)	1663(14.52)		3801(28.21)		
Time after discharge, days (IQR)	91 (173.42)		98 (153)		0.000
Examination, median, (IQR)		missing rate		missing rate	
Temperature, C	36.50 (0.40)	0.22	36.50 (0.30)	0.27	0.000
Pulse, times	78.00 (13.00)	0.1	78.00 (14.00)	0.24	0.000
SBP, mmHg	128.00 (20.00)	0.11	132.00 (27.00)	0.29	0.000
DBP, mmHg	77.00 (14.00)	0.11	80.00 (17.00)	0.29	0.000
ADA, U/L	10.70 (5.60)	0.36	12.20 (6.80)	0.84	0.000
AFP, ng/mL	2.35 (1.77)	0.52	2.21 (1.80)	0.89	0.000
ALB, g/L	38.10 (6.30)	0.18	40.40 (5.70)	0.76	0.000
ALP, U/L	82.60 (37.60)	0.19	78.00 (34.40)	0.77	0.000
ALT, U/L	19.50 (16.90)	0.15	18.40 (15.00)	0.76	0.000
APTT, seconds	27.70 (3.90)	0.25	27.90 (7.80)	0.82	0.000
AST, U/L	21.63 (11.75)	0.16	19.60 (10.40)	0.86	0.000
BA, %	0.40 (0.40)	0.2	0.40 (0.40)	0.77	0.000
CA199, U/mL	13.43 (15.71)	0.52	14.20 (14.70)	0.98	0.000
CEA, ng/mL	2.72 (2.49)	0.49	2.25 (2.38)	0.87	0.000
CK, U/L	64.00 (55.70)	0.19	68.30 (59.00)	0.87	0.000
CYFRA21-1, ng/mL	2.57 (1.86)	0.61	2.70 (1.75)	0.95	0.000
Ca, mmol/L	2.25 (0.17)	0.16	2.27 (0.19)	0.9	0.000
Cl, mmol/L	104.40 (4.50)	0.17	104.00 (4.70)	0.75	0.000
Cr, umol/L	70.90 (31.60)	0.71	67.00 (31.05)	0.75	0.000
DBIL, umol/L	3.80 (2.50)	0.2	2.80 (2.20)	0.78	0.000
DD2, mg/L	0.48 (0.92)	0.4	0.45 (0.83)	0.82	0.000
EO, %	1.83 (2.20)	0.2	1.80 (2.10)	0.78	0.000
FIB, g/L	2.93 (1.32)	0.34	3.13 (1.28)	0.82	0.000
FT3, pmol/L	4.15 (1.09)	0.64	4.22 (1.03)	0.92	0.000
FT4, pmol/L	16.30 (3.59)	0.64	16.00 (3.90)	0.92	0.000
GGT, U/L	27.50 (29.50)	0.19	26.00 (26.20)	0.76	0.000
GLB, g/L	26.50 (6.40)	0.25	25.80 (6.40)	0.77	0.000
GLU, mmol/L	6.19 (2.90)	0.19	6.84 (3.72)	0.76	0.000
HBDH, U/L	125.00 (41.00)	0.2	139.80 (48.00)	0.91	0.000
HBsAb, COI	10.09 (93.98)	0.62	5.46 (55.57)	0.95	0.000
HCT, %	37.99 (7.95)	0.2	38.50 (7.70)	0.82	0.000
HDL-C, mmol/L	1.01 (0.36)	0.2	1.14 (0.44)	0.88	0.000
HGB, g/L	126.00 (28.00)	0.2	129.00 (28.00)	0.79	0.000
HbA1c, %	7.00 (1.90)	0.61	7.30 (2.40)	0.89	0.000
K, mmol/L	3.91 (0.55)	0.17	3.94 (0.53)	0.75	0.000
LDH, U/L	198.00 (67.00)	0.2	182.00 (61.00)	0.84	0.000
LDL-C, mmol/L	2.57 (1.20)	0.2	2.53 (1.29)	0.88	0.000
Lpa, mg/L	151.00 (271.00)	0.2	111.00 (180.00)	0.93	0.000
LY, %	26.10 (14.60)	0.2	25.10 (14.50)	0.79	0.000
MCH, pg	30.30 (2.40)	0.2	30.50 (2.40)	0.8	0.000
MCHC, g/L	332.00 (15.00)	0.2	332.00 (19.25)	0.77	0.000
MCV, fl	91.20 (6.70)	0.2	91.10 (6.30)	0.78	0.000
MO, %	7.50 (3.35)	0.2	6.70 (3.00)	0.77	0.000
MPV, fl	10.70 (1.70)	0.21	10.40 (1.90)	0.78	0.000
Mg, mmol/L	0.85 (0.12)	0.34	0.87 (0.15)	0.88	0.000
NE, %	62.51 (15.80)	0.2	64.00 (14.80)	0.78	0.000
NSE, ng/mL	16.79 (7.61)	0.59	10.86 (9.45)	0.94	0.000
Na, mmol/L	140.00 (3.70)	0.17	140.10 (4.00)	0.75	0.000
PCT, %	18.00 (12.00)	0.21	0.20 (0.08)	0.83	0.000
PDW, %	13.60 (4.50)	0.21	16.00 (2.82)	0.81	0.000
PLT, 10^9/L	185.00 (89.00)	0.2	187.00 (89.00)	0.8	0.000
PT, seconds	11.90 (1.30)	0.25	11.70 (1.80)	0.8	0.000
PT-INR	1.03 (0.12)	0.29	0.98 (0.12)	0.88	0.000
Phos, mmol/L	1.14 (0.27)	0.2	1.10 (0.26)	0.78	0.000
RBC, 10^12/L	4.17 (0.94)	0.2	4.21 (0.92)	0.79	0.000
RBP, mg/L	36.40 (17.80)	0.36	38.40 (18.40)	0.92	0.000
RDW-CV, %	13.40 (1.89)	0.21	13.20 (1.95)	0.89	0.000
SG	1.01 (0.01)	0.25	1.02 (0.01)	0.8	0.000
TBIL, umol/L	10.90 (6.50)	0.18	10.55 (7.00)	0.76	0.000
TC, mmol/L	4.14 (1.61)	0.2	4.28 (1.55)	0.87	0.000
TG, mmol/L	1.33 (0.98)	0.2	1.48 (1.07)	0.99	0.000
TP, g/L	64.80 (8.70)	0.18	66.30 (8.92)	0.76	0.000
TSH, mIU/L	2.16 (2.04)	0.64	2.00 (1.88)	0.92	0.000
TT, seconds	17.60 (1.60)	0.34	17.20 (1.80)	0.8	0.000
UA, umol/L	315.00 (135.00)	0.2	316.00 (135.00)	0.76	0.000
Urea, mmol/L	5.91 (2.86)	0.15	7.26 (8.68)	0.98	0.000
WBC, 10^9/L	6.08 (2.84)	0.2	6.20 (2.78)	0.77	0.000

SBP, systolic blood pressure; DBP, diastolic blood pressure; ADA, adenosine deaminase; AFP, alpha-fetoprotein; ALB, albumin; ALP, alkaline phosphatase; ALT, alanine aminotransferase; APTT, activated partial thromboplastin time; AST, aspartate aminotransferase; BA, basophil; CA199, carbohydrate antigen 199; CEA, carcinoembryonic antigen; CK, creatine kinase; CYFRA21-1, cytokeratin-19 fragment; Cr, creatinine; DBIL, direct bilirubin; DD2, D-Dimer; EO, eosinophils; FIB, fibrinogen; FT3, free triiodothyronine; FT4, free thyroxine; GGT, gamma-glutamyl transferase; GLB, globulin; GLU, glucose; HBDH, hydroxybutyrate dehydrogenase; HBsAb, hepatitis B surface antibody; HCT, hematocrit; HDL-C, high-density lipoprotein cholesterol; HGB, hemoglobin; HbA1c, glycosylated hemoglobin; LDH, lactate dehydrogenase; LDL-C, low-density lipoprotein cholesterol; Lpa, lipoprotein(a); LY, lymphocytes; MCH, mean corpuscular hemoglobin; MCHC, mean corpuscular hemoglobin concentration; MCV, mean corpuscular volume; MO, monocytes; MPV, mean platelet colume; NE, neutrophils; NSE, neuron-specific enolase; PCT, platelet crit; PDW, platelet distribution width; PLT, platelet count; PT, prothrombin time; PT-INR, prothrombin pime international normalized ratio; Phos, phosphate; RBC, red blood cell count; RBP, retinol-binding protein; RDW-CV, red cell distribution width-coefficient of variation; SG, specific gravity; TBIL, total bilirubin; TC, total cholesterol; TG, triglycerides; TP, total protein; TSH, thyroid-stimulating hormone; TT, thrombin time; UA, uric acid; Urea, blood urea nitrogen; WBC, white blood cell count.

Table A2: Descriptive result of the case-control cohorts from NHIP, GHS, and PHS

	NHIP			GHS-M			GHS-L			TCM-HS			PHS		
	case \\ (n=5445)	control \\ (n=16335)	p-value	case \\ (n=347)	control \\ (n=1041)	p-value	case \\ (n=595)	control \\ (n=1785)	p-value	case \\ (n=852)	control \\ (n=2556)	p-value	case \\ (n=286)	control \\ (n=858)	p-value
Patients, N	3678	13813		263	968		464	1543		703	2350		203	825	
Gender															
Female, N (%)	1901 (51.69)	5764 (41.73)	0.000	137 (52.09)	449 (46.38)	0.116	201 (43.32)	575 (37.27)	0.022	400 (56.90)	996 (42.38)	0.000	119 (58.62)	437 (52.97)	0.171
Male, N (%)	1777 (48.31)	8049 (58.27)		126 (47.91)	519 (53.62)		263 (56.68)	968 (62.73)		303 (43.10)	1354 (57.62)		84 (41.38)	388 (47.03)	
Age, Year (IQR)	68 (17)	66 (18)	0.000	66 (17)	66 (16)	0.915	67 (17)	66 (14)	0.007	70 (14)	67 (16)	0.000	69 (12)	70 (12)	0.814
Examination, median, (IQR)															
Temperature, C	36.50 (0.30)	36.50 (0.30)	0.072	36.50 (0.30)	36.50 (0.20)	0.268	36.50 (0.40)	36.50 (0.30)	0.982	36.50 (0.30)	36.50 (0.30)	0.982	36.50 (0.40)	36.50 (0.30)	0.110
Pulse, times	78.00 (14.00)	78.00 (14.00)	0.000	77.50 (16.00)	79.00 (16.00)	0.019	78.00 (15.00)	78.00 (13.00)	0.106	78.00 (15.00)	79.00 (16.00)	0.004	76.00 (15.00)	76.00 (14.00)	0.695
SBP, mmHg	133.00 (29.00)	133.00 (26.00)	0.277	137.00 (26.00)	140.00 (27.50)	0.282	135.00 (27.00)	133.00 (24.00)	0.035	134.00 (25.00)	137.00 (24.00)	0.004	133.00 (27.00)	138.00 (21.00)	0.001
DBP, mmHg	79.00 (16.00)	80.00 (16.00)	0.061	78.00 (14.00)	79.00 (16.00)	0.356	77.00 (17.00)	78.00 (16.00)	0.047	77.00 (15.00)	80.00 (14.00)	0.000	80.00 (13.00)	80.00 (13.00)	0.812
GLU, mmol/L	6.33 (3.08)	6.84 (3.51)	0.000	6.29 (2.89)	7.34 (3.70)	0.011	6.02 (2.61)	6.33 (3.15)	0.084	6.27 (2.94)	7.53 (4.39)	0.000	7.23 (3.24)	7.95 (4.02)	0.341
HbA1c, %	6.90 (1.90)	7.30 (2.40)	0.000	6.82 (2.13)	7.95 (2.94)	0.000	7.00 (1.63)	7.10 (2.15)	0.457	6.70 (1.70)	7.30 (2.80)	0.000	7.50 (2.20)	7.35 (2.62)	0.839
LDL-C, mmol/L	2.42 (1.36)	2.53 (1.33)	0.010	2.25 (1.28)	2.39 (1.22)	0.565	-	-	-	2.39 (1.27)	2.44 (1.09)	0.834	2.49 (1.38)	2.79 (1.17)	0.021
HDL-C, mmol/L	1.19 (0.44)	1.14 (0.45)	0.000	1.06 (0.42)	1.07 (0.37)	0.403	-	-	-	1.27 (0.35)	1.21 (0.35)	0.003	1.10 (0.34)	1.17 (0.49)	0.261
TC, mmol/L	4.13 (1.62)	4.36 (1.62)	0.002	4.04 (1.54)	4.15 (1.47)	0.452	-	-	-	4.16 (1.60)	4.54 (1.63)	0.005	4.07 (1.66)	4.44 (1.51)	0.005
ADA, U/L	11.40 (6.50)	12.20 (6.80)	0.000	-	11.82 (83.59)	-	11.85 (6.35)	11.70 (5.85)	0.200	9.30 (5.93)	11.70 (6.80)	0.000	14.40 (7.30)	16.18 (12.36)	0.728
AFP, ng/mL	2.30 (1.70)	2.38 (1.82)	0.284	1.70 (1.62)	1.77 (1.62)	0.680	1.85 (1.50)	1.97 (1.80)	0.813	2.50 (1.60)	2.50 (2.01)	0.935	2.69 (0.81)	2.14 (1.11)	0.482
ALB, g/L	40.50 (5.30)	40.50 (6.30)	0.878	40.20 (5.65)	40.40 (4.93)	0.891	39.50 (4.90)	40.00 (4.70)	0.065	42.60 (6.00)	42.22 (6.90)	0.758	41.95 (5.30)	42.20 (7.85)	0.522
ALP, U/L	73.60 (31.35)	78.00 (35.00)	0.000	77.00 (25.50)	76.50 (30.00)	0.473	70.10 (29.20)	73.25 (30.55)	0.002	70.00 (29.00)	82.00 (38.00)	0.000	74.78 (28.30)	79.00 (21.23)	0.030
ALT, U/L	18.00 (13.20)	18.20 (15.00)	0.142	20.00 (15.00)	21.00 (17.00)	0.403	16.20 (11.50)	18.00 (14.65)	0.000	19.00 (12.00)	21.00 (13.00)	0.033	20.55 (12.00)	17.90 (15.80)	0.343
APTT, seconds	29.10 (9.10)	27.30 (6.69)	0.000	31.25 (5.50)	30.90 (4.40)	0.067	26.80 (3.20)	26.30 (3.10)	0.200	36.40 (5.75)	30.80 (10.12)	0.000	28.00 (5.55)	29.80 (10.20)	0.415
AST, U/L	18.60 (9.36)	20.30 (10.60)	0.000	-	-	-	18.00 (8.85)	21.10 (11.88)	0.000	16.00 (6.00)	21.50 (13.50)	0.003	18.60 (8.40)	17.04 (7.83)	0.112
BA, %	0.40 (0.40)	0.40 (0.40)	0.031	0.40 (0.40)	0.30 (0.30)	0.011	0.40 (0.35)	0.40 (0.30)	0.166	0.50 (0.40)	0.40 (0.40)	0.001	0.30 (0.30)	0.40 (0.30)	0.615
CA199, U/mL	13.00 (12.45)	14.36 (14.58)	0.436	-	-	-	-	-	-	9.85 (21.02)	13.80 (12.28)	0.492	5.95 (9.11)	12.04 (11.64)	0.279
CEA, ng/mL	2.05 (1.82)	2.40 (2.48)	0.000	2.46 (1.41)	2.81 (2.16)	0.437	1.46 (1.60)	1.89 (1.94)	0.000	2.08 (2.03)	2.11 (1.64)	0.969	1.75 (1.66)	2.65 (2.36)	0.267
CK, U/L	67.00 (57.00)	69.00 (57.45)	0.607	65.00 (38.00)	74.50 (59.75)	0.371	61.00 (43.75)	67.50 (61.75)	0.081	71.00 (56.00)	76.00 (53.73)	0.632	66.88 (38.40)	69.00 (57.74)	0.513
CYFRA21-1, ng/mL	2.70 (1.46)	2.73 (1.86)	0.827	3.76 (2.17)	3.00 (2.17)	0.057	-	-	-	2.50 (1.15)	2.40 (1.00)	0.146	-	3.03 (1.73)	-
Ca, mmol/L	2.27 (0.19)	2.26 (0.19)	0.652	2.25 (0.17)	2.25 (0.18)	0.556	-	-	-	2.29 (0.24)	2.28 (0.21)	0.767	2.33 (0.23)	2.39 (0.17)	0.283
Cl, mmol/L	104.20 (4.60)	104.00 (4.60)	0.127	104.77 (4.78)	104.00 (5.87)	0.208	104.05 (4.66)	104.40 (4.74)	0.041	104.80 (4.15)	104.00 (4.00)	0.010	103.70 (3.85)	102.30 (3.80)	0.029
Cr, umol/L	66.00 (29.00)	69.00 (36.00)	0.000	60.00 (27.80)	62.00 (35.80)	0.706	65.00 (26.60)	66.00 (32.00)	0.529	65.90 (27.65)	67.31 (28.55)	0.452	64.00 (26.00)	66.20 (25.70)	0.485
DBIL, umol/L	2.60 (1.94)	2.90 (2.20)	0.000	3.65 (1.70)	3.70 (2.40)	0.705	2.40 (1.50)	2.70 (1.80)	0.000	2.05 (1.47)	2.62 (1.91)	0.000	4.23 (2.29)	3.40 (2.57)	0.050
DD2, mg/L	0.42 (0.70)	0.45 (0.83)	0.027	0.40 (0.60)	0.38 (0.50)	0.889	0.47 (1.04)	0.49 (0.96)	0.688	0.37 (0.42)	0.36 (0.55)	0.579	0.32 (0.52)	0.30 (0.96)	0.838
EO, %	1.90 (2.00)	1.80 (2.10)	0.073	1.70 (1.73)	1.70 (2.00)	0.760	2.00 (2.10)	2.00 (2.40)	0.638	1.95 (2.00)	1.60 (1.88)	0.048	2.05 (1.65)	1.80 (2.15)	0.677
FIB, g/L	3.10 (0.98)	3.14 (1.25)	0.011	3.04 (0.75)	2.99 (0.92)	0.523	3.00 (1.00)	3.00 (1.20)	0.847	3.21 (0.90)	3.28 (1.01)	0.098	3.49 (0.52)	3.02 (0.85)	0.070
FT3, pmol/L	4.12 (0.94)	4.34 (1.13)	0.000	4.19 (0.94)	4.15 (0.81)	0.474	4.07 (0.93)	4.08 (0.97)	0.897	4.18 (1.12)	4.79 (1.01)	0.027	4.19 (0.69)	4.48 (0.93)	0.678
FT4, pmol/L	16.30 (3.74)	16.30 (3.57)	0.751	16.14 (3.11)	15.80 (3.37)	0.049	16.80 (3.20)	16.50 (3.40)	0.095	16.91 (3.29)	17.88 (3.59)	0.683	14.41 (6.08)	13.91 (5.55)	0.263
GGT, U/L	23.00 (21.00)	26.40 (26.50)	0.000	20.00 (17.50)	25.00 (26.00)	0.002	23.05 (18.72)	26.30 (25.90)	0.001	23.00 (21.00)	27.00 (24.00)	0.000	26.90 (20.59)	26.00 (32.15)	0.808
GLB, g/L	25.10 (5.80)	25.70 (6.40)	0.000	24.60 (5.40)	24.95 (5.92)	0.743	25.40 (5.90)	26.30 (6.30)	0.007	23.90 (5.41)	26.50 (7.50)	0.000	25.00 (5.79)	28.09 (5.90)	0.003
HBDH, U/L	135.00 (38.00)	137.00 (45.00)	0.576	-	-	-	136.00 (36.00)	142.00 (49.00)	0.222	133.50 (38.30)	132.75 (43.75)	0.320	131.00 (36.00)	128.65 (48.50)	1.000
HBsAb, COI	7.44 (51.29)	3.75 (57.82)	0.963	-	-	-	3.38 (30.46)	6.78 (67.88)	0.489	12.60 (40.10)	20.60 (168.60)	0.732	43.40 (135.03)	14.62 (62.87)	1.000
HCT, %	38.70 (6.80)	38.40 (8.00)	0.937	-	-	-	38.60 (7.40)	38.80 (7.50)	0.715	38.70 (5.75)	40.40 (9.45)	0.000	41.45 (4.95)	40.70 (6.00)	0.390
HGB, g/L	129.00 (24.00)	128.00 (28.00)	0.592	127.00 (23.50)	131.00 (27.00)	0.167	129.00 (27.50)	129.00 (28.00)	0.118	128.00 (22.00)	133.00 (31.00)	0.002	135.50 (18.00)	135.00 (19.50)	0.550
K, mmol/L	3.91 (0.54)	3.97 (0.55)	0.000	3.82 (0.54)	3.90 (0.50)	0.024	3.91 (0.53)	3.96 (0.53)	0.059	3.88 (0.52)	4.03 (0.51)	0.000	4.02 (0.47)	4.07 (0.45)	0.555
LDH, U/L	179.00 (59.00)	179.00 (60.75)	0.821	-	-	-	186.00 (60.00)	188.00 (61.00)	0.055	163.00 (47.00)	170.00 (52.00)	0.007	155.88 (37.59)	165.70 (51.05)	0.815
Lpa, mg/L	98.00 (208.00)	98.00 (160.00)	0.054	115.55 (217.47)	89.65 (180.75)	0.057	324.30 (134.50)	-	-	88.00 (161.50)	111.00 (184.50)	0.289	123.73 (105.16)	279.52 (242.73)	0.005
LY, %	26.20 (13.00)	24.40 (16.13)	0.000	27.15 (13.98)	26.00 (14.93)	0.925	26.50 (12.30)	26.30 (13.70)	0.582	28.02 (13.65)	25.15 (13.18)	0.007	28.30 (10.90)	26.55 (11.05)	0.306
MCH, pg	30.60 (2.40)	30.50 (2.30)	0.185	30.30 (2.05)	30.30 (2.10)	0.423	30.90 (2.50)	30.40 (2.30)	0.000	30.50 (2.35)	30.30 (2.18)	0.214	30.80 (1.55)	30.50 (1.80)	0.191
MCHC, g/L	332.00 (19.00)	330.50 (18.00)	0.098	230.00 (16.00)	232.00 (19.00)	0.099	337.00 (14.00)	332.00 (12.00)	0.000	330.00 (14.25)	248.00 (95.25)	0.000	333.00 (12.00)	333.00 (13.00)	0.844
MCV, fl	91.60 (6.00)	91.50 (6.45)	0.193	91.40 (5.36)	90.20 (5.73)	0.012	91.40 (6.00)	91.40 (6.03)	0.098	92.30 (6.80)	91.90 (6.64)	0.588	93.15 (4.57)	91.40 (5.00)	0.036
MO, %	6.90 (2.70)	6.70 (3.30)	0.010	7.40 (3.30)	7.00 (3.05)	0.018	6.80 (2.30)	6.80 (2.65)	0.860	8.00 (2.62)	6.50 (2.97)	0.000	5.70 (2.50)	5.90 (2.40)	0.566
MPV, fl	10.30 (1.60)	10.50 (1.70)	0.002	11.10 (1.50)	11.05 (1.81)	0.702	10.07 (1.51)	10.10 (1.60)	0.451	10.30 (1.43)	10.20 (1.97)	0.723	9.75 (2.50)	10.00 (2.20)	0.628
Mg, mmol/L	0.89 (0.14)	0.87 (0.14)	0.000	0.84 (0.11)	0.80 (0.13)	0.002	0.84 (0.12)	0.83 (0.12)	0.097	0.95 (0.11)	0.95 (0.14)	0.708	-	-	-
NE, %	63.00 (13.55)	63.90 (15.50)	0.021	63.35 (15.73)	63.40 (17.50)	0.804	62.90 (12.80)	63.10 (14.93)	0.914	61.30 (12.53)	65.15 (14.48)	0.000	62.50 (11.80)	63.40 (12.05)	0.790
NSE, ng/mL	9.02 (8.95)	10.82 (8.67)	0.000	11.60 (4.86)	12.00 (3.46)	0.436	12.82 (5.12)	14.60 (6.75)	0.008	3.46 (1.17)	4.00 (4.87)	0.000	-	10.34 (1.79)	-
Na, mmol/L	140.40 (3.98)	140.10 (4.00)	0.012	140.00 (5.11)	139.00 (4.98)	0.025	140.50 (3.80)	139.70 (3.80)	0.000	140.50 (3.50)	140.00 (3.50)	0.008	141.10 (2.85)	141.00 (3.05)	0.693
PCT, %	0.20 (0.07)	0.20 (0.08)	0.461	0.20 (0.07)	0.20 (0.08)	0.641	0.20 (0.08)	0.19 (0.08)	0.115	0.18 (0.07)	0.20 (0.08)	0.262	0.18 (0.08)	0.18 (0.08)	0.258
PDW, %	16.00 (2.60)	15.80 (4.00)	0.000	14.70 (3.47)	15.70 (3.30)	0.067	16.10 (0.80)	16.20 (0.60)	0.018	13.30 (5.45)	16.40 (0.50)	0.000	16.45 (1.73)	16.10 (3.20)	0.002
PLT, 10^9/L	190.00 (83.50)	189.00 (91.00)	0.510	175.00 (66.67)	179.00 (75.50)	0.627	198.00 (81.00)	191.50 (89.00)	0.054	186.00 (88.00)	178.00 (80.00)	0.070	173.00 (79.50)	157.00 (29.00)	0.427
PT, seconds	11.90 (2.00)	11.50 (1.70)	0.000	11.20 (1.13)	11.20 (1.10)	0.397	11.20 (1.20)	11.10 (1.20)	0.007	13.10 (1.12)	12.30 (1.60)	0.000	12.00 (1.65)	12.00 (1.60)	0.578
PT-INR	0.98 (0.11)	0.99 (0.13)	0.679	-	-	-	0.98 (0.10)	0.96 (0.11)	0.010	-	0.96 (0.14)	-	0.96 (0.18)	0.93 (0.09)	0.904
Phos, mmol/L	1.08 (0.24)	1.11 (0.27)	0.000	1.16 (0.29)	1.18 (0.29)	0.252	1.11 (0.23)	1.13 (0.23)	0.006	0.99 (0.22)	1.04 (0.21)	0.000	1.01 (0.25)	1.15 (0.21)	0.385
RBC, 10^12/L	4.21 (0.79)	4.19 (0.94)	0.546	4.19 (0.84)	4.33 (0.97)	0.217	4.22 (0.83)	4.23 (0.87)	0.747	4.17 (0.69)	4.32 (1.04)	0.003	4.78 (0.41)	4.70 (0.82)	0.786
RBP, mg/L	39.62 (17.25)	40.00 (18.68)	0.940	32.40 (12.50)	32.30 (14.75)	0.841	36.80 (30.50)	43.35 (33.05)	0.446	44.65 (15.88)	43.60 (20.25)	0.302	70.00 (0.00)	34.10 (20.45)	1.000
RDW-CV, %	13.00 (1.50)	13.30 (2.00)	0.000	12.70 (0.92)	12.70 (1.20)	0.971	-	-	-	12.90 (1.30)	13.00 (1.70)	0.071	12.50 (1.10)	12.40 (1.20)	0.401
SG	1.02 (0.01)	1.02 (0.01)	0.000	1.02 (0.01)	1.02 (0.01)	0.002	1.02 (0.01)	1.02 (0.01)	0.000	1.01 (0.01)	1.02 (0.01)	0.000	1.02 (0.00)	1.02 (0.01)	0.219
TBIL, umol/L	10.20 (6.50)	10.40 (6.80)	0.056	11.00 (5.26)	11.60 (6.62)	0.112	9.40 (6.30)	10.70 (6.90)	0.000	9.66 (6.37)	11.93 (7.09)	0.000	13.47 (6.03)	11.38 (6.71)	0.121
TG, mmol/L	2.01 (1.18)	1.44 (0.81)	0.059	-	-	-	-	-	-	-	-	-	1.41 (0.52)	1.45 (0.41)	0.983
TP, g/L	65.60 (8.40)	66.40 (9.34)	0.008	65.50 (8.40)	65.30 (7.40)	0.899	64.70 (7.90)	66.00 (8.50)	0.005	66.27 (8.02)	68.65 (11.17)	0.000	67.25 (7.26)	70.17 (8.22)	0.012
TSH, mIU/L	2.02 (1.90)	2.03 (2.00)	0.702	2.17 (1.72)	2.45 (2.42)	0.352	1.89 (1.61)	1.95 (1.74)	0.184	1.67 (2.15)	2.10 (2.48)	0.843	2.33 (0.75)	2.40 (2.58)	0.976
TT, seconds	17.30 (1.80)	17.10 (1.60)	0.007	14.55 (1.20)	14.80 (1.50)	0.113	17.40 (1.20)	17.40 (1.40)	0.438	17.85 (1.60)	16.90 (1.38)	0.000	15.55 (1.22)	15.10 (1.47)	0.229
UA, umol/L	317.00 (129.30)	319.00 (137.75)	0.363	300.50 (118.00)	312.00 (139.33)	0.240	323.00 (130.00)	326.00 (141.75)	0.891	317.00 (132.00)	306.00 (131.50)	0.487	315.00 (110.43)	345.00 (142.00)	0.453
Urea, mmol/L	6.99 (4.40)	10.74 (17.21)	0.000	-	-	-	6.98 (4.50)	7.39 (6.14)	0.020	-	-	-	4.29 (0.35)	7.94 (0.86)	0.333
WBC, 10^9/L	6.10 (2.36)	6.15 (2.66)	0.587	5.97 (2.00)	6.23 (2.85)	0.434	6.30 (2.50)	6.10 (2.60)	0.145	6.21 (2.18)	6.38 (2.79)	0.379	5.94 (2.41)	6.16 (2.21)	0.953

SBP, systolic blood pressure; DBP, diastolic blood pressure; ADA, adenosine deaminase; AFP, alpha-fetoprotein; ALB, albumin; ALP, alkaline phosphatase; ALT, alanine aminotransferase; APTT, activated partial thromboplastin time; AST, aspartate aminotransferase; BA, basophil; CA199, carbohydrate antigen 199; CEA, carcinoembryonic antigen; CK, creatine kinase; CYFRA21-1, cytokeratin-19 fragment; Cr, creatinine; DBIL, direct bilirubin; DD2, D-Dimer; EO, eosinophils; FIB, fibrinogen; FT3, free triiodothyronine; FT4, free thyroxine; GGT, gamma-glutamyl transferase; GLB, globulin; GLU, glucose; HBDH, hydroxybutyrate dehydrogenase; HBsAb, hepatitis B surface antibody; HCT, hematocrit; HDL-C, high-density lipoprotein cholesterol; HGB, hemoglobin; HbA1c, glycosylated hemoglobin; LDH, lactate dehydrogenase; LDL-C, low-density lipoprotein cholesterol; Lpa, lipoprotein(a); LY, lymphocytes; MCH, mean corpuscular hemoglobin; MCHC, mean corpuscular hemoglobin concentration; MCV, mean corpuscular volume; MO, monocytes; MPV, mean platelet colume; NE, neutrophils; NSE, neuron-specific enolase; PCT, platelet crit; PDW, platelet distribution width; PLT, platelet count; PT, prothrombin time; PT-INR, prothrombin pime international normalized ratio; Phos, phosphate; RBC, red blood cell count; RBP, retinol-binding protein; RDW-CV, red cell distribution width-coefficient of variation; SG, specific gravity; TBIL, total bilirubin; TC, total cholesterol; TG, triglycerides; TP, total protein; TSH, thyroid-stimulating hormone; TT, thrombin time; UA, uric acid; Urea, blood urea nitrogen; WBC, white blood cell count.

Table A3: Description of the T2DM patients discharge EHRs from PHSs.

	PHSs // (n=12661)	
Total patients, N	8907	
Gender		
Female, N (%)	4817 (54.08)	
Male, N (%)	4090 (45.92)	
Age, Year (IQR)	70 (12)	
Depression or anxiety rate per 1000 p	22.11	
Time after discharge, days (IQR)	93 (152)	
Examination, median, (IQR)		missing rate
Temperature, C	36.50 (0.40)	0.28
Pulse, times	76.00 (14.00)	0.22
SBP, mmHg	137.00 (24.00)	0.19
DBP, mmHg	80.00 (13.00)	0.19
ADA, U/L	12.70 (7.70)	0.94
AFP, ng/mL	2.10 (1.36)	0.97
ALB, g/L	42.60 (5.90)	0.81
ALP, U/L	77.42 (32.00)	0.81
ALT, U/L	18.04 (13.81)	0.81
APTT, seconds	30.10 (7.12)	0.97
AST, U/L	17.88 (9.00)	0.82
BA, %	0.30 (0.30)	0.79
CA199, U/mL	14.37 (12.79)	0.98
CEA, ng/mL	2.50 (1.98)	0.97
CK, U/L	72.06 (58.00)	0.85
CYFRA21-1, ng/mL	2.56 (1.34)	1
Ca, mmol/L	2.34 (0.25)	0.85
Cl, mmol/L	102.80 (4.00)	0.8
Cr, umol/L	66.62 (28.01)	0.8
DBIL, umol/L	3.80 (2.40)	0.81
DD2, mg/L	0.32 (1.08)	0.91
EO, %	2.00 (1.80)	0.79
FIB, g/L	3.05 (0.86)	0.94
FT3, pmol/L	4.24 (1.08)	0.96
FT4, pmol/L	13.87 (4.71)	0.95
GGT, U/L	24.40 (20.31)	0.81
GLB, g/L	26.40 (6.04)	0.82
GLU, mmol/L	7.56 (4.22)	0.82
HBDH, U/L	131.00 (39.00)	0.88
HBsAb, COI	10.14 (52.73)	0.98
HCT, %	40.30 (6.10)	0.79
HDL-C, mmol/L	1.18 (0.45)	0.83
HGB, g/L	135.00 (21.00)	0.79
HbA1c, %	7.50 (2.90)	0.89
K, mmol/L	4.06 (0.52)	0.8
LDH, U/L	163.75 (49.32)	0.98
LDL-C, mmol/L	2.49 (1.18)	0.83
Lpa, mg/L	218.94 (258.22)	0.97
LY, %	27.85 (11.80)	0.82
MCH, pg	30.60 (2.20)	0.79
MCHC, g/L	333.00 (15.00)	0.79
MCV, fl	91.60 (5.80)	0.79
MO, %	5.60 (2.50)	0.79
MPV, fl	10.00 (2.00)	0.79
Mg, mmol/L	-	1
NE, %	63.10 (12.10)	0.79
NSE, ng/mL	10.34 (1.64)	1
Na, mmol/L	140.50 (3.80)	0.8

PCT, %	0.19 (0.08)	0.79
PDW, %	16.30 (1.00)	0.79
PLT, 10^9/L	173.00 (74.50)	0.97
PT, seconds	11.80 (1.64)	0.92
PT-INR	0.96 (0.15)	0.94
Phos, mmol/L	1.09 (0.20)	0.98
RBC, 10^12/L	4.43 (0.66)	0.97
RBP, mg/L	36.25 (14.47)	1
RDW-CV, %	12.50 (1.30)	0.82
SG	1.02 (0.01)	0.85
TBIL, umol/L	12.40 (6.90)	0.81
TC, mmol/L	4.40 (1.59)	0.81
TG, mmol/L	1.31 (0.79)	0.98
TP, g/L	69.10 (8.19)	0.81
TSH, mIU/L	2.19 (1.82)	0.98
TT, seconds	15.30 (1.93)	0.95
UA, umol/L	304.00 (127.05)	0.82
Urea, mmol/L	5.54 (2.13)	0.99
WBC, 10^9/L	6.20 (2.44)	0.79
