# Supplementary material for: Applying contrastive pre-training for depression and anxiety risk prediction in type 2 diabetes patients based on heterogeneous electronic health records: a primary healthcare case study
Source: J Am Med Inform Assoc. 2023 Dec 7;31(2):445–55. doi: 10.1093/jamia/ocad228 (PMC10797279; doi:10.1093/jamia/ocad228)
Supplement: ocad228_Supplementary_Data [file ocad228_supplementary_data.zip › ocad228_Supplementary_Data/Appendice_2.pdf]

# 1. Appendices

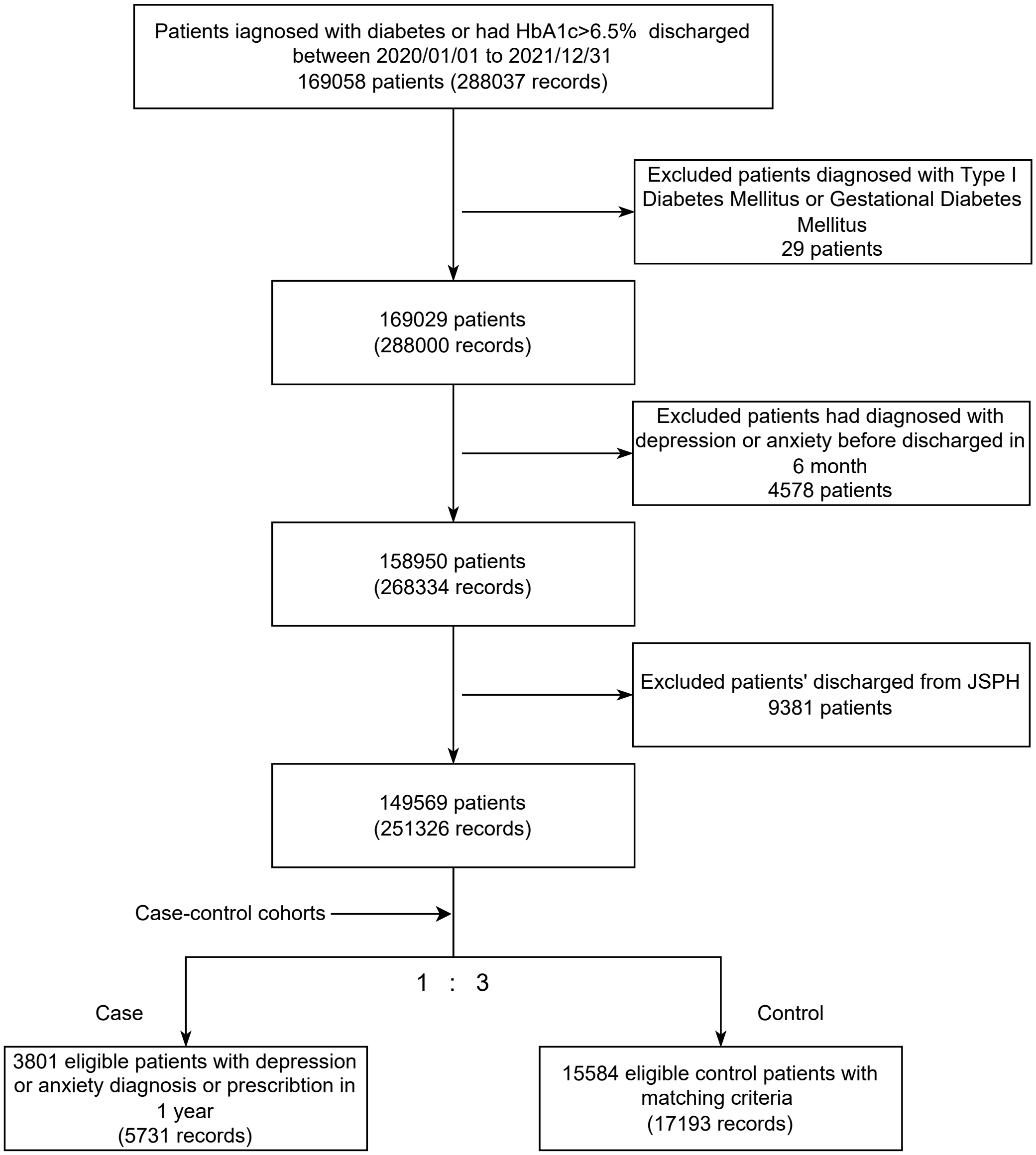


Figure A1: Flowchart for the construction of a case-control cohort of discharged patients with T2DM from NHIP

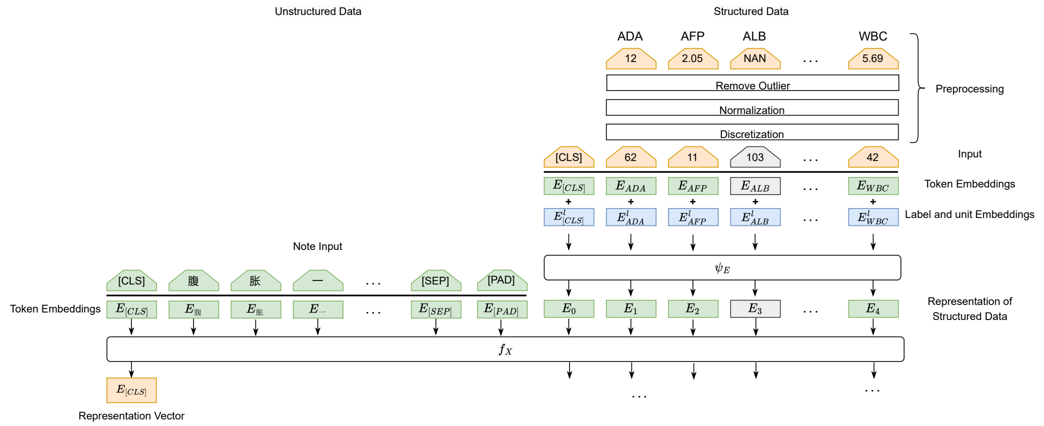


Figure A2: The fusion procedure of structured and unstructured data from discharge document.

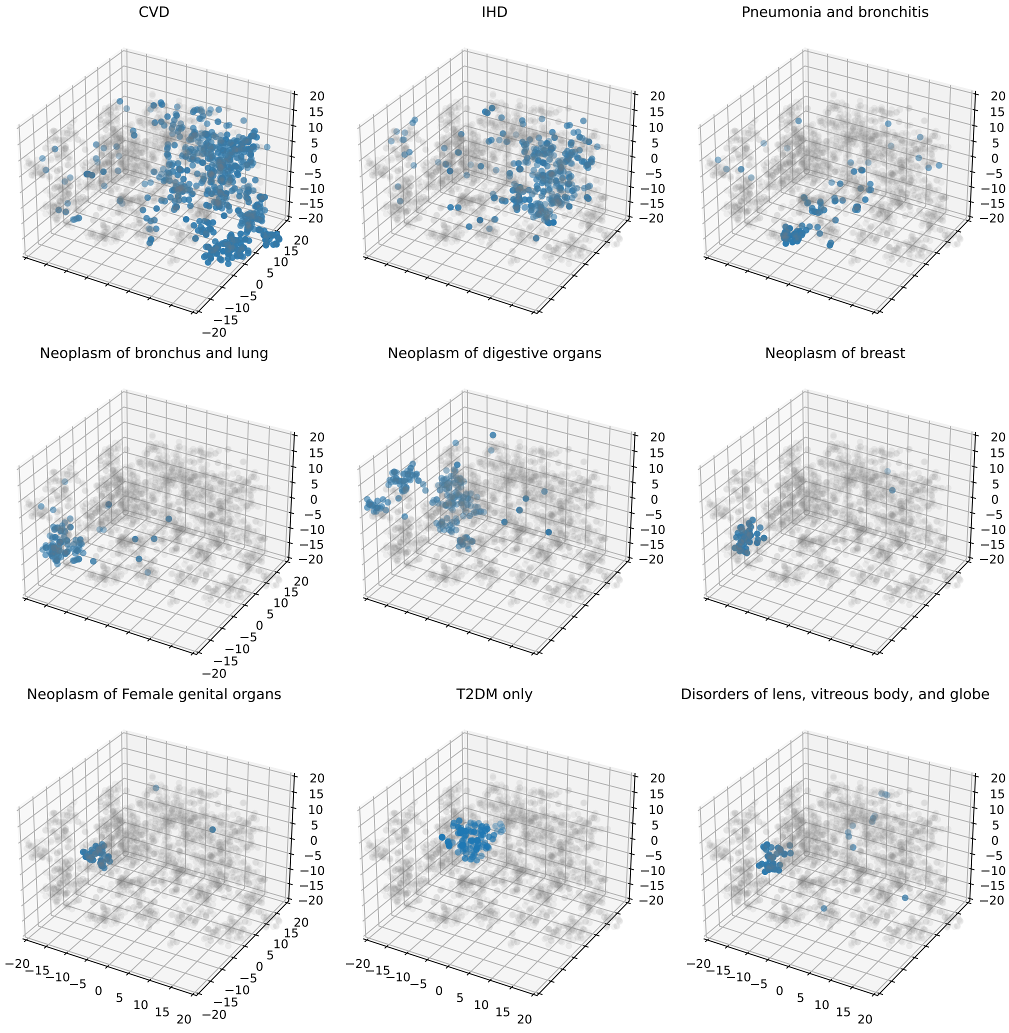


Figure A3: After applying the EHR pre-training model to the discharge records in NHIP, clustering of certain diseases was observed. The solid spots in each sub-figures reffed to the positive record of each disease.

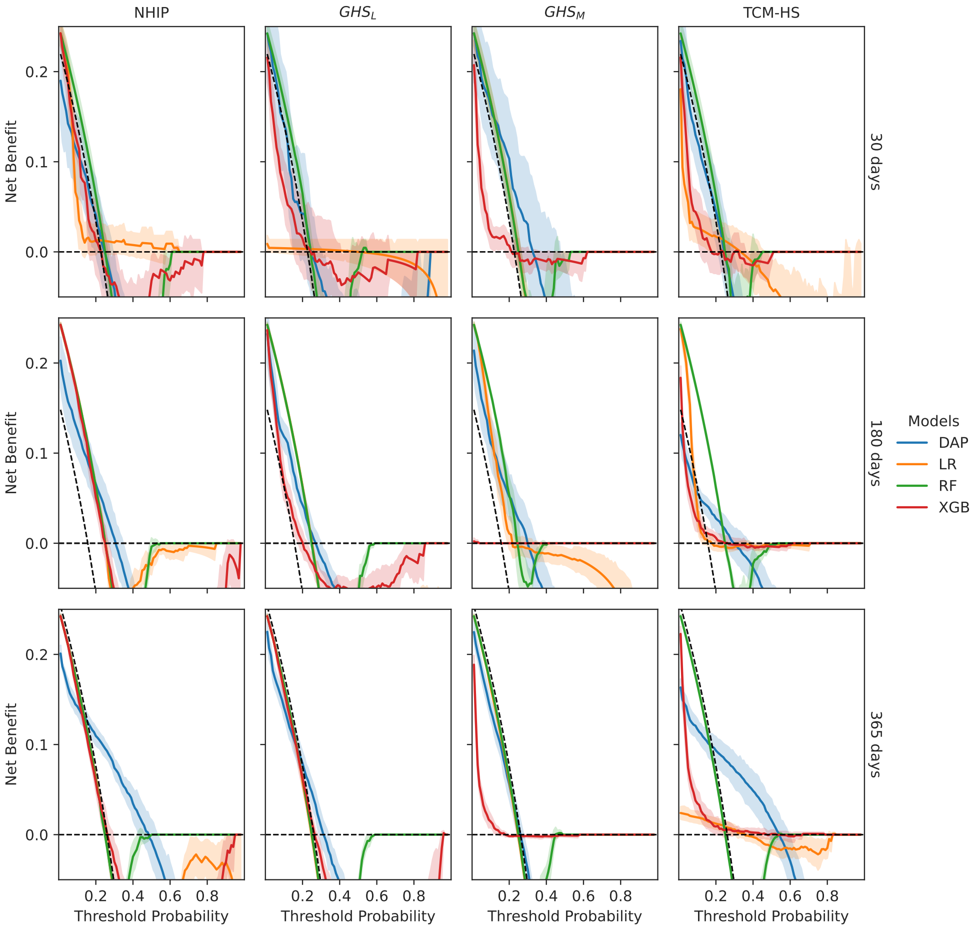


Figure A4: The decision curve analysis based on training with data from different healthcare services, including NHIP, $GHS_L$, $GHS_M$, TCM-HS, and conducted 10 external validations using records from PHS. The last column represents the results of the 10-fold validation on PHS.

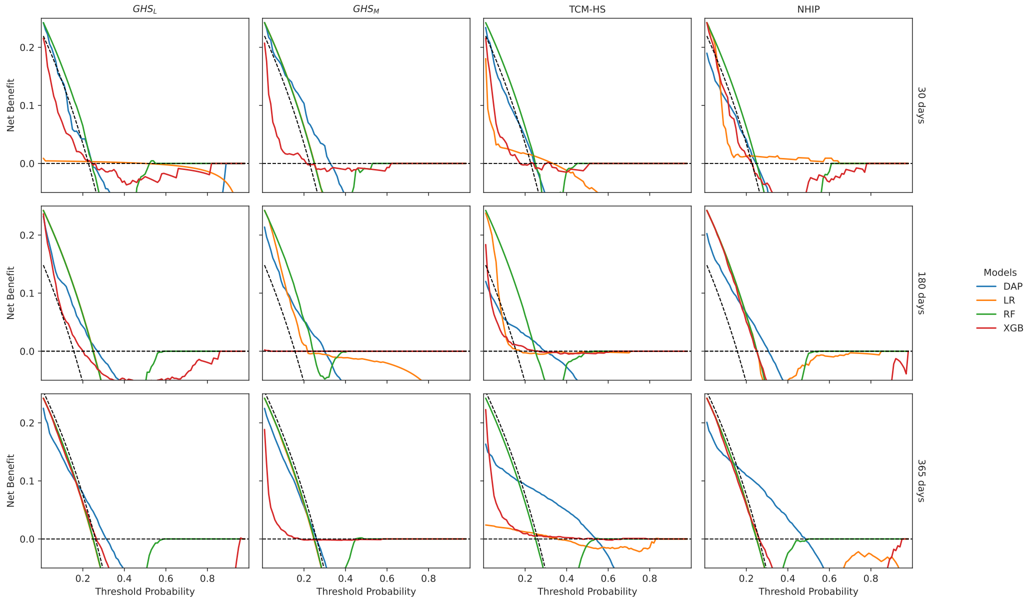


Figure A5: The decision curve analysis based on training with data from different healthcare services, including NHIP, $GHS_L$, $GHS_M$, TCM-HS, and conducted validation using all records from PHS cohort.

Table A4: Internal validation result of NHIP and PHS on 30-days, 180-days, 365-days post-discharge prediction tasks.

| | 30 days | | | | 180 days | | | | 365 days | | | |
|---|---|---|---|---|---|---|---|---|---|---|---|---|
| | ROC-AUC | p-value | PR-AUC | p-value | ROC-AUC | p-value | PR-AUC | p-value | ROC-AUC | p-value | PR-AUC | p-value |
| NHIP† | | | | | | | | | | | | |
| LR | 0.70±0.035 | 0.000 | 0.46±0.050 | 0.000 | 0.63±0.019 | 0.000 | 0.41±0.024 | 0.000 | 0.60±0.011 | 0.000 | 0.32±0.009 | 0.000 |
| RF | 0.81±0.022 | 0.006 | 0.63±0.040 | 0.002 | 0.77±0.014 | 0.000 | 0.57±0.025 | 0.000 | 0.73±0.011 | 0.000 | 0.51±0.015 | 0.000 |
| XGB | 0.82±0.016 | 0.008 | 0.65±0.035 | 0.010 | 0.77±0.012 | 0.000 | 0.58±0.019 | 0.000 | 0.72±0.012 | 0.000 | 0.46±0.026 | 0.000 |
| DAP | 0.85±0.029 | ref | 0.70±0.043 | ref | 0.82±0.010 | ref | 0.67±0.011 | ref | 0.80±0.010 | ref | 0.61±0.018 | ref |
| PHS | | | | | | | | | | | | |
| LR | 0.51±0.100 | 0.007 | 0.29±0.112 | 0.155 | 0.53±0.057 | 0.000 | 0.28±0.055 | 0.000 | 0.60±0.072 | 0.000 | 0.41±0.101 | 0.000 |
| RF | 0.52±0.097 | 0.010 | 0.32±0.112 | 0.287 | 0.47±0.076 | 0.000 | 0.27±0.079 | 0.000 | 0.60±0.057 | 0.000 | 0.34±0.056 | 0.000 |
| XGB | 0.55±0.111 | 0.042 | 0.39±0.124 | 0.981 | 0.52±0.081 | 0.000 | 0.29±0.074 | 0.000 | 0.65±0.072 | 0.000 | 0.41±0.072 | 0.000 |
| DAP | 0.66±0.118 | ref | 0.40±0.185 | ref | 0.73±0.039 | ref | 0.54±0.083 | ref | 0.91±0.028 | ref | 0.80±0.067 | ref |

†The records in PHS were removed.
Evaluation metrics included ROC-AUC and PR-AUC. We conducted a t-test to compare the differences in results between the two groups generated from the 10-fold data.

Table A5: External validation result of NHIP, GHS-L, GHS-M, and TCM-HS on 30-days, 180-days, 365-days post-discharge prediction tasks.

| | 30 days | | | | 180 days | | | | 365 days | | | |
|---|---|---|---|---|---|---|---|---|---|---|---|---|
| | ROC-AUC | p-value | PR-AUC | p-value | ROC-AUC | p-value | PR-AUC | p-value | ROC-AUC | p-value | PR-AUC | p-value |
| NHIP† | | | | | | | | | | | | |
| LR | 0.49±0.114 | 0.819 | 0.31±0.111 | 0.401 | 0.44±0.044 | 0.000 | 0.23±0.037 | 0.003 | 0.51±0.076 | 0.000 | 0.30±0.074 | 0.000 |
| RF | 0.48±0.140 | 0.998 | 0.27±0.116 | 0.948 | 0.49±0.069 | 0.001 | 0.25±0.046 | 0.022 | 0.52±0.061 | 0.000 | 0.28±0.058 | 0.000 |
| XGB | 0.48±0.126 | 0.938 | 0.28±0.121 | 0.733 | 0.50±0.066 | 0.002 | 0.26±0.049 | 0.088 | 0.53±0.065 | 0.000 | 0.26±0.039 | 0.000 |
| DAP | 0.48±0.159 | ref | 0.27±0.102 | ref | 0.60±0.061 | ref | 0.31±0.063 | ref | 0.75±0.045 | ref | 0.47±0.081 | ref |
| GHS-L | | | | | | | | | | | | |
| LR | 0.44±0.184 | 0.778 | 0.27±0.105 | 0.487 | 0.46±0.066 | 0.022 | 0.23±0.037 | 0.099 | 0.50±0.055 | 0.000 | 0.28±0.060 | 0.009 |
| RF | 0.47±0.193 | 0.893 | 0.33±0.168 | 0.171 | 0.49±0.074 | 0.123 | 0.25±0.038 | 0.307 | 0.50±0.061 | 0.000 | 0.27±0.037 | 0.000 |
| XGB | 0.46±0.174 | 0.975 | 0.31±0.198 | 0.342 | 0.44±0.067 | 0.006 | 0.22±0.029 | 0.019 | 0.61±0.051 | 0.476 | 0.37±0.053 | 0.804 |
| DAP | 0.46±0.140 | ref | 0.24±0.103 | ref | 0.55±0.082 | ref | 0.27±0.050 | ref | 0.62±0.059 | ref | 0.36±0.055 | ref |
| GHS-M | | | | | | | | | | | | |
| LR | 0.44±0.113 | 0.000 | 0.26±0.089 | 0.035 | 0.51±0.122 | 0.134 | 0.29±0.101 | 0.389 | 0.40±0.069 | 0.005 | 0.23±0.042 | 0.281 |
| RF | 0.51±0.152 | 0.025 | 0.29±0.158 | 0.218 | 0.45±0.110 | 0.006 | 0.24±0.046 | 0.017 | 0.50±0.059 | 0.737 | 0.27±0.050 | 0.453 |
| XGB | 0.50±0.162 | 0.022 | 0.26±0.084 | 0.044 | 0.50±0.071 | 0.018 | 0.26±0.054 | 0.102 | 0.49±0.063 | 0.848 | 0.27±0.037 | 0.473 |
| DAP | 0.64±0.086 | ref | 0.38±0.154 | ref | 0.59±0.077 | ref | 0.33±0.099 | ref | 0.49±0.060 | ref | 0.26±0.053 | ref |
| TCM-HS | | | | | | | | | | | | |
| LR | 0.49±0.149 | 0.810 | 0.32±0.115 | 0.273 | 0.50±0.075 | 0.204 | 0.25±0.055 | 0.049 | 0.56±0.058 | 0.000 | 0.33±0.053 | 0.000 |
| RF | 0.53±0.116 | 0.586 | 0.33±0.115 | 0.167 | 0.48±0.119 | 0.187 | 0.26±0.072 | 0.107 | 0.55±0.056 | 0.000 | 0.30±0.036 | 0.000 |
| XGB | 0.46±0.116 | 0.443 | 0.28±0.144 | 0.688 | 0.49±0.062 | 0.115 | 0.26±0.036 | 0.044 | 0.56±0.066 | 0.000 | 0.31±0.066 | 0.000 |
| DAP | 0.50±0.137 | ref | 0.26±0.117 | ref | 0.55±0.091 | ref | 0.31±0.066 | ref | 0.74±0.035 | ref | 0.46±0.073 | ref |

†The records in PHS were removed.
Evaluation metrics included ROC-AUC and PR-AUC. We conducted a t-test to compare the differences in results between the two groups generated from the 10-fold data.

Table A6: Internal validation result of NHIP and PHS on 30-days, 180-days, 365-days post-discharge prediction tasks (LDA methods for baselines).

| | 30 days | | | | 180 days | | | | 365 days | | | |
|---|---|---|---|---|---|---|---|---|---|---|---|---|
| | ROC-AUC | p-value | PR-AUC | p-value | ROC-AUC | p-value | PR-AUC | p-value | ROC-AUC | p-value | PR-AUC | p-value |
| NHIP† | | | | | | | | | | | | |
| LR | 0.69±0.016 | 0.000 | 0.45±0.036 | 0.000 | 0.62±0.014 | 0.000 | 0.38±0.018 | 0.000 | 0.58±0.012 | 0.000 | 0.31±0.017 | 0.000 |
| RF | 0.73±0.026 | 0.000 | 0.50±0.032 | 0.000 | 0.68±0.016 | 0.000 | 0.45±0.022 | 0.000 | 0.65±0.012 | 0.000 | 0.38±0.008 | 0.000 |
| XGB | 0.74±0.017 | 0.000 | 0.53±0.039 | 0.000 | 0.70±0.014 | 0.000 | 0.48±0.019 | 0.000 | 0.66±0.013 | 0.000 | 0.37±0.018 | 0.000 |
| DAP | 0.85±0.029 | ref | 0.70±0.043 | ref | 0.82±0.010 | ref | 0.67±0.011 | ref | 0.80±0.010 | ref | 0.61±0.018 | ref |
| PHS | | | | | | | | | | | | |
| LR | 0.53±0.099 | 0.016 | 0.30±0.115 | 0.188 | 0.53±0.053 | 0.000 | 0.27±0.048 | 0.000 | 0.59±0.070 | 0.000 | 0.39±0.088 | 0.000 |
| RF | 0.51±0.203 | 0.060 | 0.37±0.188 | 0.786 | 0.59±0.110 | 0.000 | 0.41±0.112 | 0.000 | 0.72±0.083 | 0.000 | 0.54±0.108 | 0.000 |
| XGB | 0.57±0.126 | 0.104 | 0.40±0.191 | 0.942 | 0.63±0.068 | 0.000 | 0.43±0.066 | 0.000 | 0.71±0.077 | 0.000 | 0.52±0.115 | 0.000 |
| DAP | 0.66±0.118 | ref | 0.40±0.185 | ref | 0.73±0.039 | ref | 0.54±0.083 | ref | 0.91±0.028 | ref | 0.80±0.067 | ref |

†The records in PHS were removed.
Evaluation metrics included ROC-AUC and PR-AUC. We conducted a t-test to compare the differences in results between the two groups generated from the 10-fold data.

Table A7: External validation result of NHIP, GHS-L, GHS-M, and TCM-HS on 30-days, 180-days, 365-days post-discharge prediciton tasks (LDA methods for baselines).

| | 30 days | | | | 180 days | | | | 365 days | | | |
|---|---|---|---|---|---|---|---|---|---|---|---|---|
| | ROC-AUC | p-value | PR-AUC | p-value | ROC-AUC | p-value | PR-AUC | p-value | ROC-AUC | p-value | PR-AUC | p-value |
| NHIP† | | | | | | | | | | | | |
| LR | 0.42±0.000 | 0.296 | 0.21±0.000 | 0.083 | 0.41±0.000 | 0.000 | 0.20±0.000 | 0.000 | 0.52±0.000 | 0.000 | 0.27±0.000 | 0.000 |
| RF | 0.52±0.000 | 0.439 | 0.39±0.000 | 0.002 | 0.49±0.000 | 0.000 | 0.27±0.000 | 0.070 | 0.63±0.000 | 0.000 | 0.39±0.000 | 0.009 |
| XGB | 0.41±0.000 | 0.207 | 0.19±0.000 | 0.028 | 0.45±0.000 | 0.000 | 0.21±0.000 | 0.000 | 0.56±0.000 | 0.000 | 0.29±0.000 | 0.000 |
| DAP | 0.48±0.159 | ref | 0.27±0.102 | ref | 0.60±0.061 | ref | 0.31±0.063 | ref | 0.75±0.045 | ref | 0.47±0.081 | ref |
| GHS-L | | | | | | | | | | | | |
| LR | 0.24±0.000 | 0.000 | 0.15±0.000 | 0.015 | 0.45±0.000 | 0.002 | 0.21±0.000 | 0.002 | 0.48±0.000 | 0.000 | 0.26±0.000 | 0.000 |
| RF | 0.52±0.000 | 0.242 | 0.21±0.000 | 0.402 | 0.49±0.000 | 0.035 | 0.27±0.000 | 0.867 | 0.55±0.000 | 0.001 | 0.31±0.000 | 0.011 |
| XGB | 0.61±0.000 | 0.004 | 0.25±0.000 | 0.678 | 0.63±0.000 | 0.004 | 0.33±0.000 | 0.001 | 0.58±0.000 | 0.029 | 0.30±0.000 | 0.002 |
| DAP | 0.46±0.140 | ref | 0.24±0.103 | ref | 0.55±0.082 | ref | 0.27±0.050 | ref | 0.62±0.059 | ref | 0.36±0.055 | ref |
| GHS-M | | | | | | | | | | | | |
| LR | 0.54±0.000 | 0.001 | 0.22±0.000 | 0.003 | 0.70±0.000 | 0.000 | 0.43±0.000 | 0.004 | 0.34±0.000 | 0.000 | 0.19±0.000 | 0.001 |
| RF | 0.38±0.000 | 0.000 | 0.18±0.000 | 0.000 | 0.46±0.000 | 0.000 | 0.22±0.000 | 0.004 | 0.51±0.000 | 0.300 | 0.28±0.000 | 0.240 |
| XGB | 0.56±0.000 | 0.009 | 0.25±0.000 | 0.012 | 0.55±0.000 | 0.174 | 0.29±0.000 | 0.265 | 0.64±0.000 | 0.000 | 0.42±0.000 | 0.000 |
| DAP | 0.64±0.086 | ref | 0.38±0.154 | ref | 0.59±0.077 | ref | 0.33±0.099 | ref | 0.49±0.060 | ref | 0.26±0.053 | ref |
| TCM-HS | | | | | | | | | | | | |
| LR | 0.47±0.000 | 0.923 | 0.20±0.000 | 0.256 | 0.49±0.000 | 0.005 | 0.22±0.000 | 0.000 | 0.64±0.000 | 0.000 | 0.36±0.000 | 0.000 |
| RF | 0.27±0.000 | 0.000 | 0.15±0.000 | 0.014 | 0.51±0.000 | 0.033 | 0.22±0.000 | 0.000 | 0.60±0.000 | 0.000 | 0.48±0.000 | 0.490 |
| XGB | 0.69±0.000 | 0.000 | 0.33±0.000 | 0.032 | 0.55±0.000 | 0.394 | 0.34±0.000 | 0.479 | 0.62±0.000 | 0.000 | 0.41±0.000 | 0.007 |
| DAP | 0.47±0.113 | ref | 0.25±0.112 | ref | 0.58±0.086 | ref | 0.32±0.071 | ref | 0.77±0.041 | ref | 0.49±0.083 | ref |

†The records in PHS were removed.
Evaluation metrics included ROC-AUC and PR-AUC. We conducted a t-test to compare the differences in results between the two groups generated from the 10-fold data.
